# TriCON: A Carbon‐Based Triple‐Modal Nanoplatform for Pancreatic Cancer Therapy

**DOI:** 10.1002/advs.202512978

**Published:** 2026-01-18

**Authors:** Xinyu Peng, Jiaxing Huang, Shengnan Lv, Jian Zhang, Liangliang Zhang, Huan Liu, Yan Liu, Zhen‐An Qiao, Feng Wei, Bao‐Lian Su

**Affiliations:** ^1^ Department of Hepatobiliary and Pancreatic Surgery General Surgery Center The First Hospital of Jilin University Changchun P. R. China; ^2^ State Key Laboratory of Inorganic Synthesis and Preparative Chemistry College of Chemistry Jilin University Changchun P. R. China; ^3^ Key Laboratory of Jilin Province For Zoonosis Prevention and Control Institute of Military Veterinary Medicine Academy of Military Medical Sciences Changchun P. R. China; ^4^ State Key Laboratory of Pathogen and Biosecurity Changchun Veterinary Research Institute Chinese Academy of Agricultural Sciences Changchun P. R. China; ^5^ State Key Laboratory of Advanced Technology For Materials Synthesis and Processing Wuhan University of Technology Wuhan P. R. China; ^6^ Laboratory of Inorganic Materials Chemistry (CMI) University of Namur Namur Belgium

**Keywords:** chemo‐immunotherapy, gene editing, pancreatic cancer, poliovirus receptor, TriCON

## Abstract

Pancreatic cancer, recognized for its high malignancy and tumor immunosuppressive microenvironment, has been refractory to conventional therapeutic modalities, necessitating the exploration of novel treatment strategies. Among these, reprogramming of the tumor immunosuppressive microenvironment is a promising strategy to enhance the efficacy of tumor immunotherapy. The CRISPR/Cas9 system‐based gene editing further offers a viable approach for precise regulation of endogenous gene expression associated with tumor immunosuppression. Current delivery vectors face a trilemma between biosafety profiles, expansion capacity, and targeting accuracy. To this end, we developed a triple‐modality therapeutic platform, termed TriCON (Triple Convergent Oncology Nanotherapy), characterized by three core mechanistic attributes: spatiotemporal convergence, stimuli‐responsive controllability, and tumor‐microenvironment modulatory conductivity. This orchestrated combination of rationally designed gene editing (targeting poliovirus receptor), nano‐encapsulated doxorubicin (DOX) chemotherapy, and checkpoint blockade immunotherapy demonstrated enhanced synergistic antitumor activity in pancreatic ductal adenocarcinoma (PDAC) models, achieving tumor regression through enhanced chemotherapy, immunogenic cell death induction, and natural killer (NK) cells activation. The platform achieved superior in vivo gene editing (14.2% PVR editing efficiency) via optimized endosomal escape and CRISPR system release. This triaxial approach establishes a programmable nanotherapeutic paradigm that synergizes gene editing precision with chemo‐immunotherapy, offering a novel framework for PDAC treatment.

## Graphical abstract

We developed TriCON, a triple‐modality nanotherapeutic platform, to treat pancreatic ductal adenocarcinoma (PDAC) by synergizing gene editing, chemotherapy, and immunotherapy. TriCON utilizes CRISPR/Cas9 to target the poliovirus receptor (PVR), combined with nano‐encapsulated doxorubicin and checkpoint blockade. This approach achieved significant tumor regression in PDAC models through enhanced chemotherapy, immunogenic cell death, and NK cell activation, establishing a promising programmable nanotherapeutic paradigm.

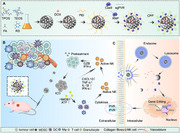

## 1 Introduction

As the most aggressive form of gastrointestinal cancer, pancreatic ductal adenocarcinoma (PDAC) always lacks effective treatment options [1]. Strategies to promote tumor regression by enhancing protective immune responses have received much attention. In recent years, immune checkpoint therapy has made significant advancements, particularly with monoclonal antibodies (mAbs) targeting programmed cell death protein 1/programmed cell death 1 ligand 1 (PD‐1/PD‐L1) [2] and cytotoxic T lymphocyte‐associated protein 4 (CTLA‐4) [3]. These therapies have achieved remarkable progress in treating various types of cancers, including Hodgkin's lymphoma [4], melanoma [5], and non‐small cell lung cancer [6]. However, only a small proportion of PDAC patients respond to current tumor immunotherapy [7]. Active exploration of tactics to reprogram immunosuppressive tumors into immune‐activated tumors potentially enhances the efficacy of immune checkpoint therapy in PDAC.

Unlike the adaptive immunity represented by T cells, natural killer (NK) cells exhibit cytotoxic activity against target cells without prior antigen sensitization [8]. Simultaneously, NK cell therapy does not trigger the emergence of graft‐versus‐host disease (GVHD), thereby, allogeneic NK cells provide an opportunity for the advancement of NK cell therapeutic strategies as a turnkey product for CAR‐NK therapy and utilized in B cell tumors [9], hepatocellular carcinoma [10], and glioblastoma [11] as an adjuvant therapy. Recently, a novel checkpoint axis, the poliovirus receptor (PVR) and the T cell immunoreceptor with immunoglobulin (Ig) and immunoreceptor tyrosine‐based inhibitory motif domain (TIGIT) was identified as a promising target for immunological intervention [12]. The blockade of the TIGIT/PVR axis has been shown to reverse NK cell exhaustion and enhance anti‐tumor efficacy in a variety of cancers, including hepatocellular carcinoma [13], breast cancer [14], and pancreatic cancer [15]. Furthermore, PVR is highly expressed in numerous cancers and promotes invasion, migration, proliferation, and angiogenesis [16]. PVR knockdown has been demonstrated to inhibit tumor growth and reduce metastatic load in various mouse tumor models.

Microbial adaptive immune systems, consisting of clusters of regularly interspaced short palindromic ‐repeats (CRISPR) and RNA‐guided Cas9 nucleases (CRISPR‐associated) from bacteria and archaea, have been found as a stable and versatile platform for effective genome editing in eukaryotic cells [17]. In clinical applications, well‐designed genetic modifications could result in enduring therapeutic effects and may potentially cure specific diseases. We therefore hypothesized that CRISPR/Cas9‐based blockade of PVR in tumor cells would serve as a permanent strategy to evoke more effective and durable antitumor immunity. The CRISPR/Cas9 system can be applied by using different forms of biomolecules: pDNA, mRNA/sgRNA, or Cas9/sgRNA ribonucleoproteins (RNP) [18]. Among these, the pre‐assembled RNP is often regarded as a straightforward and effective approach. Nevertheless, the considerable size of the Cas9 protein and the instability of the sgRNA in vitro represent the big challenges to the effective intracellular delivery of RNP. Recently, Novel nanoparticle delivery systems, such as liposomes [19], copper sulfide [20], gold nanoparticles [21], organometallic frameworks [22], and polymers [23], have shown great potential for the efficient intracellular delivery of proteins. Hence, it remains highly desirable to develop a simple and efficient in vivo RNP delivery method that would make the application of this technology less costly for treating cancer patients.

**SCHEME 1:**
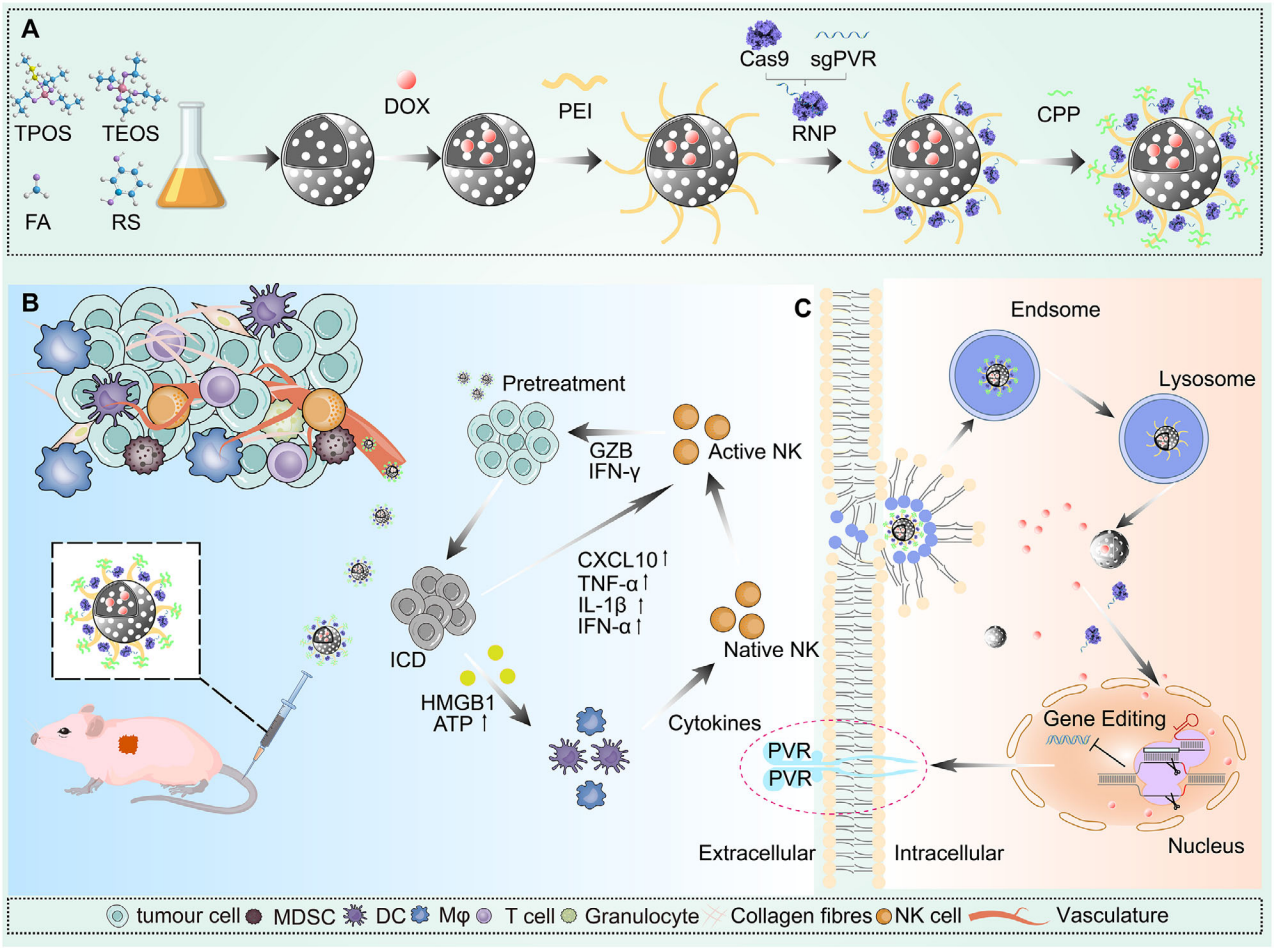
Illustration of sequential synthesis and treatment of pancreatic cancer with a nanococktail of TriCON/RNP‐PVR with spatiotemporal synergy of chemotherapy and immune checkpoints. (A) Process of preparation of TriCON/RNP‐PVR. (B) CRISPR/Cas9 strategy reprograms immunosuppressive tumor environment. (C) Illustration of PVR genome editing in tumor cells.

Herein, we have designed a triple‐modality therapeutic platform, termed TriCON (Triple Convergent Oncology Nanotherapy). This approach aims to reprogram tumor immunosuppressive microenvironment (TIME) and trigger multifaceted immune responses to enhance cancer immunotherapy. The chemical structure of TriCON and its intracellular delivery mechanism are shown in Scheme 1. TriCON features a programmable, assembled multi‐compartment structure designed for the efficient co‐loading of DOX and RNPs through π‐π interactions and electrostatic double docking. TriCON achieved controlled release of DOX through pH‐responsive, tumor‐selective activation, while preserving Cas9 activity via proton‐nucleic acid chaperoning. The molecular Velcro (polyethyleneimine/Cell‐penetrating peptide, PEI/CPP) enhances membrane penetration and facilitates proton sponge‐driven endosomal rupture, leading to the intracellular relocalization of ribonucleoproteins (RNPs). Single‐step CRISPR (TriCON/RNP) effectively penetrates the nucleus to edit the PVR, while DOX‐induced immunogenic cell death (ICD) activates anti‐tumor immune cells through the secretion of cytokines. Additionally, spatiotemporally enhanced natural killer (NK) cells directly target and eliminate tumor cells. This paper presents a novel anticancer strategy that is anticipated to be further applied in the field of tumor immunocombination gene therapy.

## 2 Results and Discussion

### 2.1 Design, Preparation, and Characterization of TriCON

Here, the carbon‐based spherical nanoparticles (CSN) were synthesized using a modified one‐pot method without surfactants [24]. To enhance surface functionalization capacity, these nanoparticles underwent wet oxidation, generating hydroxyl (‐OH) and carboxyl (‐COOH) groups on their surfaces. TriCON was constructed by first engineering these mesoporous nanoparticles, which were subsequently loaded with DOX (referred to DOX@CSN). For RNP complex preparation, nuclear‐localized recombinant Cas9 protein was complexed with synthetic sgRNAs following established procedures [25]. To optimize the gene editing efficiency, cationic PEI was integrated with the Cas9/sgRNA complex to establish charge equilibrium. The PEI‐modified complex was electrostatically conjugated to the negatively charged Cas9/sgPVR RNP, yielding DOX‐RNP‐loaded nanocomplexes. (referred to DOX@CSN‐RNP). Finally, CPP with a high affinity for tumor cells was coated onto the surface of DOX@CSN‐RNP, resulting in the formulation termed TriCON.

**FIGURE 1:**
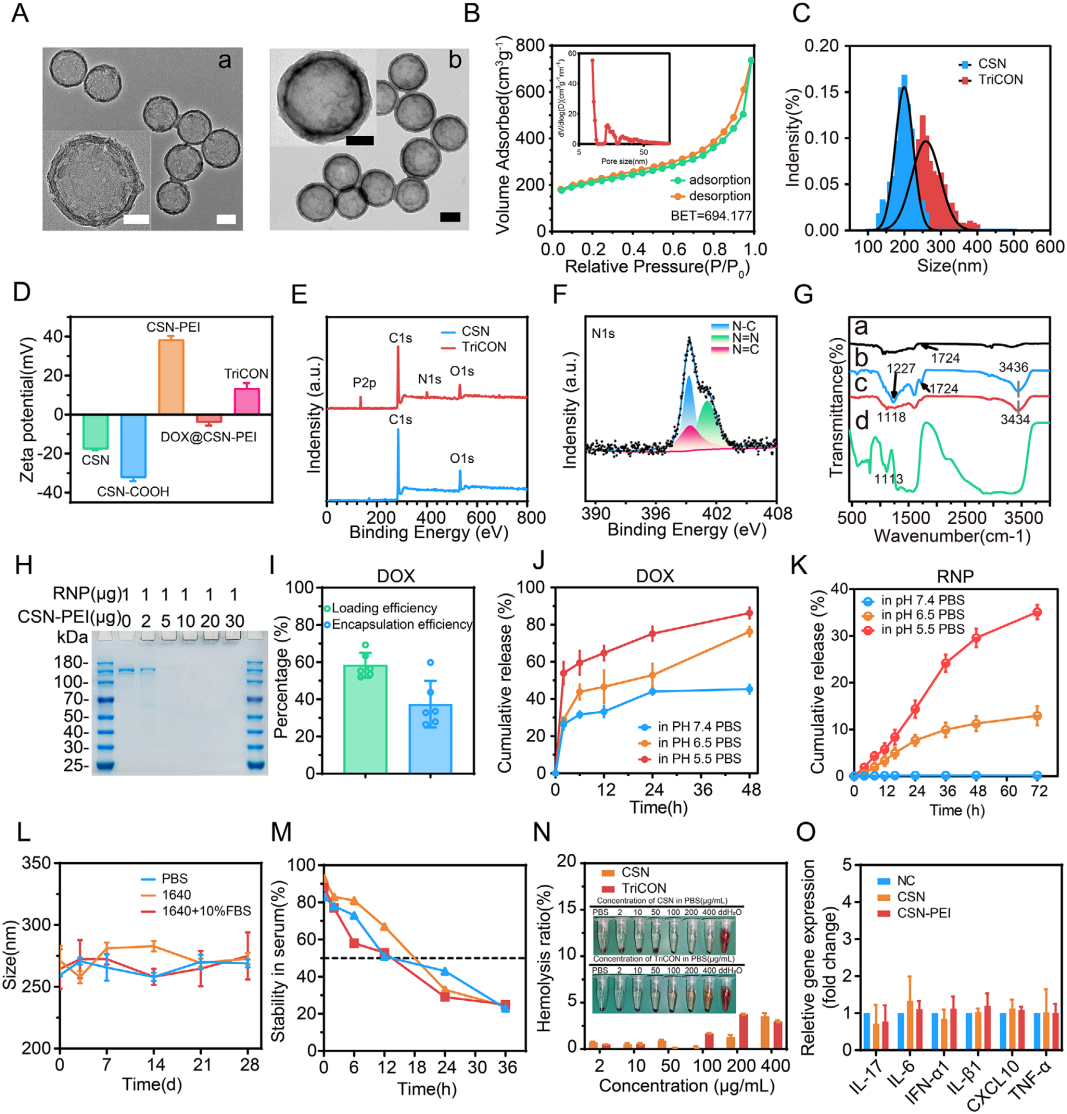
Physico‐chemical characterization of nanocomposites. (A) TEM images of CSN (a), TriCON (b) (scale bar: 100 nm; scale bar in magnified image: 50 nm). (B) The N2 sorption isotherms and (inset) pore size distribution curve of as‐synthesized CSN. (C) Particle size distributions of CSN, TriCON. (D) The zeta potential of CSN, CSN‐PEI, DOX@CSN‐PEI, and TriCON. (E) XPS spectrum of CSN, TriCON. (F) N1s of TriCON. (G) FT‐IR spectra of CSN (a), CSN‐COOH(b), CSN‐PEI(c), TriCON (d). (H) Gel electrophoretic migration of CSN‐PEI and RNP fusions with different mass ratios. (I) The drug loading efficiency and encapsulation efficiency of TriCON for DOX. (J) DOX release profile of TriCON in PBS at pH 7.4, pH 6.5 and pH 5.5 over 48 h. (K) The cumulative release of RNP by TriCON in PBS at pH 7.4, pH 6.5, and pH 5.5 over 72 h. (total RNP content set at 100%). (L) Dynamic light scattering measurements of TriCON at different time points. (M) The stability of Cas9 in the samples was assessed at various time points. N) Hemolysis rate of murine erythrocyte suspensions after incubation with different concentrations of nanoparticles. The inserted image shows the phenomenon after centrifugation. (O) mRNA levels of inflammation‐related genes in nanoparticle‐treated cells. All data are presented as mean ± SD. Data in (D), (J), (K), (N), and (O) are from *n* = 3 biologically independent samples. Data in (I) is from *n* = 6 biologically independent samples.

As shown in Figure 1A, the transmission electron microscope (TEM) image of the original CSN indicates that the primitive CSN was well‐distributed, with an average size of 196 ± 27 nm. CSN underwent a series of surface modifications, culminating in the attachment of RNP to its surface to produce TriCON nanoparticles. RNP bound to the outer layer of the TriCON surface can be observed using TEM following uranium salt staining. TEM results demonstrated that the diameter of the final nanocomplex increased to 250 nm. Furthermore, nitrogen isothermal adsorption‐desorption curves confirmed that our synthesized CSN possess a hollow mesoporous structure. The Brunauer–Emmett–Teller (BET) surface area and average pore diameter of CSN were determined to be 694.177 m^2^/g and 5 nm, respectively (Figure 1B). The cationic polymer polyethyleneimine (PEI) was covalently bonded to CSN through an amination reaction to construct cationic CSN. The CSN‐PEI was then cationically surface‐bonded with pre‐incubated Cas9‐sgRNA, which exhibited a net negative charge due to electrostatic interactions. Dynamic light scattering (DLS) measurements revealed that TriCON was formed from nanoparticles with good dispersion, a diameter of 262 ± 46 nm, and a zeta potential of +13.4 mV (Figure 1C,D). X‐ray photoelectron spectroscopy (XPS) analysis (Figure 1E) of CSN was consistent with previous literature [26]. The N1s peak and P2p peak observed at 399.12 and 134.89 eV in TriCON, respectively, indicating that the nitrogen and phosphorus content was 5.89% and 7.43% (Figure 1F). A typical signal at 1113 cm^−1^, attributed to the amide group, was observed in the Fourier transform infrared (FTIR) spectroscopy (Figure 1G), confirming the successful modification of PEI [27].

### 2.2 Drug Release Kinetics, Size Stability, and Safety of TriCON

Noting CSN‐COOH carries a negative charge (−32.34 mV) and exhibits a relatively low encapsulation efficiency of 3.2% due to repulsion caused by the negative charge of Cas9/sgRNA (Figure 1D and Figure S1A). To overcome the electrostatic repulsion, we attempted to functionalize CSN using PEI. The charge difference between CSN‐COOH and CSN‐PEI particles encapsulated by the Cas9/sgRNA complex was measured, and PEI‐functionalized CSN exhibited a significant potential shift of +38.44 mV (Figure 1D). Importantly, the PEI fusion complex enhanced its encapsulation rate in the CSN carrier to 14.33% (Figure S1B). UV spectra were further used to assess the loading and encapsulation efficiency of DOX, which were calculated to be 58.31% and 37.39%, respectively (Figure 1I). This confirms that the discharge capacity of DOX and RNP is both critical and necessary. Followingly, DOX release profiles under various pH conditions in vitro were recorded. The amount of DOX released in PBS buffer at pH 5.5 (86.25%) was significantly greater than that observed in PBS buffer at pH 7.4 (45.32%) (Figure 1J), indicating pH‐responsive drug release. Meanwhile, RNP release exhibited similar characteristics, with TriCON showing significantly increased release in pH 5.5 buffer compared to pH 7.4 buffer (Figure 1K). We proposed that the slow pH‐responsive drug release would be attributed to the protonation of the CSN‐PEI [28].

To verify the long‐term stability of TriCON, TriCON was stored in different solutions at 4 °C. The changes in the hydrodynamic diameter of TriCON in phosphate‐buffered saline (PBS), RPMI 1640, and complete medium containing 10% fetal bovine serum (FBS) were characterized using DLS, and no significant changes in color or DLS diameter were observed, indicating the good stability of TriCON (Figure 1L). Serum stability analysis further demonstrated that the sgRNA and Cas9 remained stable at over 50% after 12 h of co‐culture with 10% FBS (refer to Figure 1M for details).

CSN safety was evaluated by the hemolysis assay (Figure 1N). After incubation with erythrocytes for 2 h, the modified TriCON demonstrated no hemolytic activity. In contrast, water, serving as a positive control, caused the rupture of erythrocytes and hemolysis. To further assess the safety of TriCON on normal cells, the normal pancreatic cells (HPDE6C7) were incubated with naked CSN, CSN‐COOH, or CSN‐PEI for 48 h, then cytotoxicity assay and qPCR were conducted to detect the expression levels of cell growth and inflammation cytokines. The data demonstrated that all three agents exhibited low cytotoxicity, with the cell survival rates exceeding 85% even at concentrations of 125 and 250 µg/mL (Figure S2). Meanwhile, none of the three nanoparticles triggered cellular inflammation (Figure 1O).

### 2.3 Cell Internalization and in Vitro Cytotoxic Effect of TriCON

**FIGURE 2:**
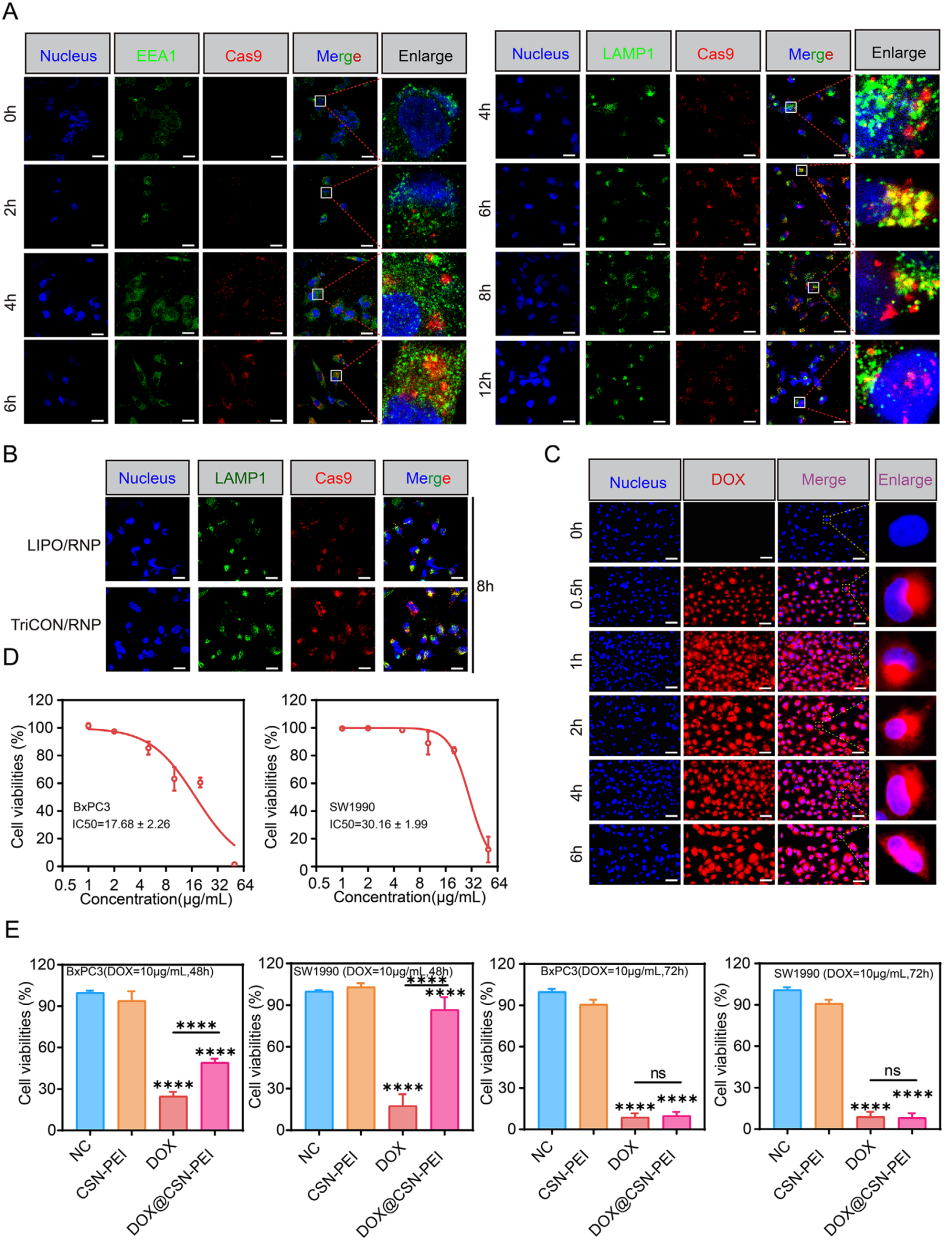
Evaluation of cytotoxicity, cellular uptake, and endosome escape ability. (A) Confocal microscope images of BxPC3 cells after incubating with TriCON/RNP for 12 h. LAMP1 labeled lysosomes in green (EEA1 labeled Endosomes in green), Alexa Fluor 594 labeled Cas9 in red, and Hoechst 33342 labeled nuclei in blue. Scale bars are 25 µm. (B) Confocal microscope images of BxPC3 cells after incubating with LIPO/RNP and CSN‐PEI/RNP for 8 h. LAMP1 labeled lysosomes in green, Alexa Fluor 594 labeled Cas9 in red, and Hoechst 33342 labeled nuclei in blue. Scale bars are 25 µm. (C) Confocal microscope images of BxPC3 cells after incubating with TriCON for 0, 0.5, 1, 2, 4, 6 h. Scale bars are 40 µm. The cellular viability of (D) The cellular viability of BxPC3 and SW1990 cells after being treated by different concentrations of DOX@CSN‐PEI after 48 h. (E) The cellular viability of BxPC3 and SW1990 cells after being treated by CSN‐PEI, DOX, and DOX@CSN‐PEI after 48 and 72 h. All data are presented as mean ± SD. Data in (D) and (E) are from *n* = 3 biologically independent samples. Statistical significance was assessed using one‐way analysis of variance (ANOVA). (ns for *p* > 0.05, ^****^ *p* < 0.0001).

To elucidate the mechanism of CSN‐mediated intracellular delivery of RNP, we investigated the cellular uptake of the CSN/RNP complex in BxPC3 cells by using fluorescent microscopy. The CSN/RNP was labeled with Alexa Fluor 594 dye, while the endosomes/lysosomes and nuclei were stained with Alexa Fluor 488 dye and Hoechst 33342, respectively. As illustrated in Figure 2A and Figure S3, CSN/RNP predominantly co‐localized with endosomes at 6 h postingestion, and with lysosomes after 8 h, then CSN/RNP nanoparticles can effectively escape from lysosomes after 12 h of incubation, suggesting their robust lysosomal escape capability under the proton sponge effect. Then we found that the red‐labeled CSN/RNP clearly entered the nucleus, indicating that the Cas9 fused with nuclear localization sequences facilitates the delivery of the Cas9/sgRNA complex to the nucleus of BxPC3 cells. Meanwhile, as compared with the CSN/RNP, which induced stronger fluorescence, the weak fluorescence of Cas9‐Alexa Fluor 594 (red) was observed in BxPC3 cells treated with LIPO/RNP (Figure 2B and Figure S4), further suggesting that the uptake of Cas9 was enhanced in CSN/RNP. Furthermore, DOX was successfully enriched within the cells and co‐localized with the nucleus after 6 h (Figure 2C).

To further confirm the cytotoxic effect of DOX@CSN‐PEI nanoparticles, BxPC3 and SW1990 cells were cultured with DOX@CSN‐PEI at the indicated time points. As shown in Figure 2D, DOX@CSN‐PEI demonstrated a dose‐dependent cytotoxic effect, with the 50% inhibitory concentrations (IC50) of DOX@CSN‐PEI at 17.68 and 30.16 µg/mL in BxPC3 and SW1990 cells, respectively. In addition, as shown in Figure 2E, the cytotoxic effect of DOX@CSN‐PEI (10 µg/mL) was significantly higher than that of CSN‐PEI control; however, it was lower than that of the 10 µg/mL free DOX treatment group for 48 h. We thought this attenuation was due to the slow release from the hollow mesoporous structure of DOX@CSN‐PEI [29]. As expected, after extending the treatment time to 72 h, no significant difference was observed between DOX@CSN‐PEI and the free DOX group.

### 2.4 Gene Disruption of PVR With CSN/RNP in Vitro

**FIGURE 3:**
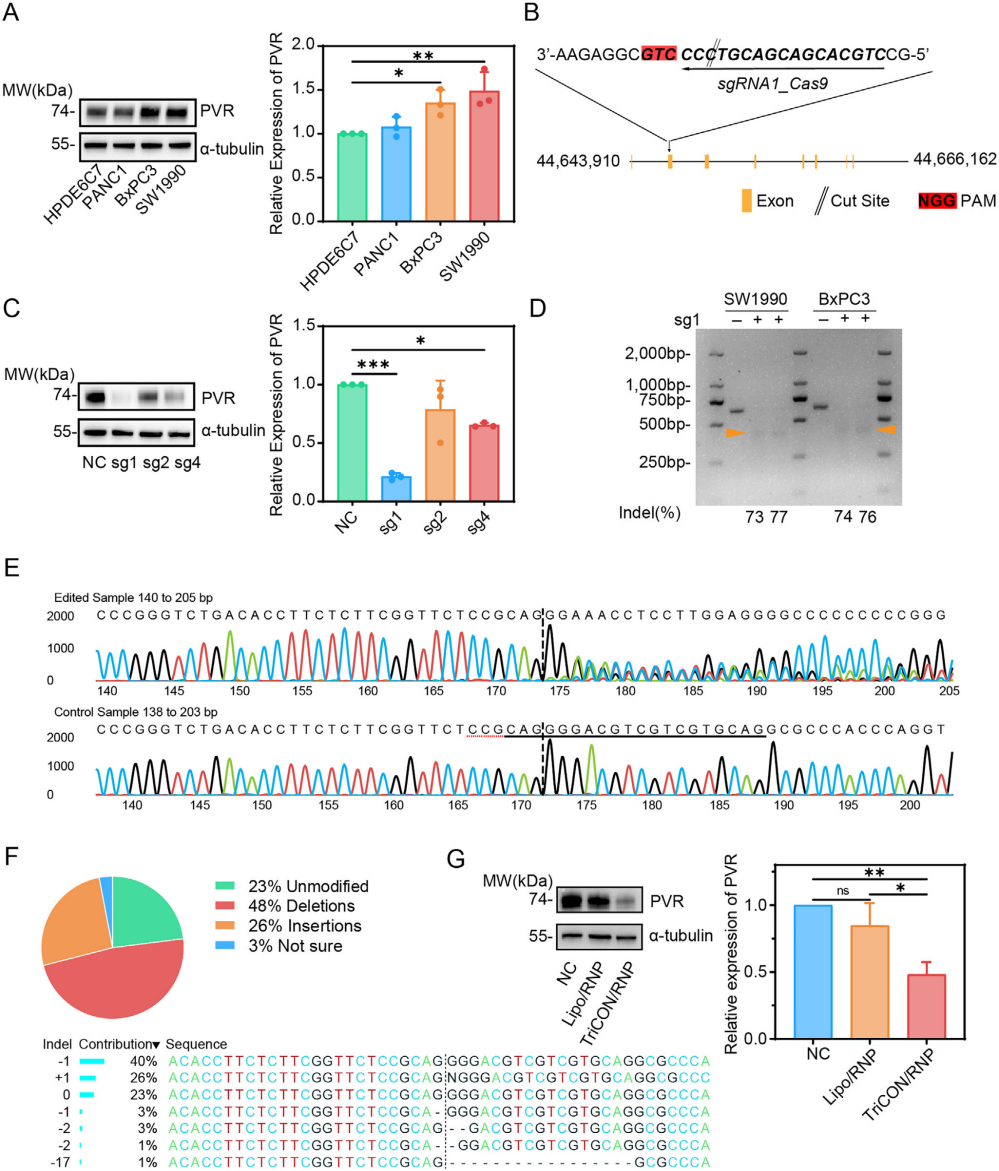
Evaluation of PVR genome editing in vitro. (A) The expression of PVR in the pancreatic normal epithelial cell line and pancreatic cancer cell lines. (B) Schematic of the target site of Pvr exon 2. (C) Quantification of PVR expression in cells treated with different sgRNAs. (D) T7E1 detection of sgRNA1 mediated cleavage of targeted PVR gene with TriCON/RNP in BxPC3 and SW1990. (E) Chromatogram of PVR genome sequencing in sg1‐treated cells. (F) ICE analysis of Sanger sequencing results. (G) PVR expression in cells based on sg1 after 48 h treatment with Lipo/RNP and TriCON/RNP. All data are presented as mean ± SD. Data in (A), (C) and (G) are from *n* = 3 biologically independent samples. Statistical significance was assessed using one‐way analysis of variance (ANOVA). (ns for *p* > 0.05, ^*^ *p* < 0.05, ^**^ *p* < 0.01, ^***^ *p* < 0.001).

To ascertain the efficacy of genome editing mediated by TriCON nanoparticles, we conducted a comparative analysis to identify the PDAC cell line with the highest PVR expression, which was subsequently utilized for gene editing experiments. As shown in Figure 3A, PVR expression was significantly upregulated in pancreatic cancer cell lines, particularly in SW1990. Figure 3B illustrates the targeting and cleavage sites of RNPs on the genome, which typically occur at the third base upstream of the protospacer adjacent motif (PAM) sequence. Next, several candidate sequences for sgRNA were designed to evaluate the selective gene targeting efficiency of human PVR (Figure S5A). The sgRNA1, sgRNA2, and sgRNA3 were selected due to their superior enzymatic efficiency compared to the other sgRNA candidates. Notably, the Cas9/sgRNA1 complex demonstrated high editing efficiency in the cleavage assay of the 205 and 411 bp fragments containing the PVR target sequence (Figure S5B,C).

We then validated the knockout efficiency of the CRISPR system in cells. After incubating with TriCON/RNP‐PVR for 48 h, the expression of PVR was examined by western‐blot, and the results demonstrated that all three sgRNAs suppressed the level of PVR, and sgRNA1 showed an excellent knockout effect (Figure 3C). In addition, the frequency insertion and deletion (indel) assessment was performed by using the T7 nucleic acid endonuclease I (T7E1) assay to evaluate the genome editing efficiency at the PVR genomic locus. As shown in Figure 3D and Figure S6A, incubation with RNP alone resulted in a negligible mutation frequency, whereas a significantly higher mutation frequency (>70%) was observed in cells treated with TriCON/RNP‐PVR. Furthermore, Sanger sequencing analysis of polymerase chain reaction (PCR) amplicons from the TriCON/RNP‐PVR treatment group provided strong evidence for the mutations at the PVR locus. As control, free RNP did not induce these targeted mutations (Figure 3E, Figure S6B). Based on the sequencing data, Inference of CRISPR Edits (ICE) was utilized to evaluate the efficacy of PVR gene disruption, in which the total disruption efficiency of TriCON/RNP‐PVR was 77%, including 48% of deletion mutations and 26% of insertion mutations (Figure 3F). In addition, western‐blot analysis of PVR expression level further demonstrated that the TriCON/RNP‐PVR‐treated group was more efficient than the liposome group knockout, thereby confirming the potential of the TriCON‐loaded genome editing system in vitro (Figure 3G).

### 2.5 PVR Knockout Promotes the Cytotoxic Effect of NK Cells In Vitro

**FIGURE 4:**
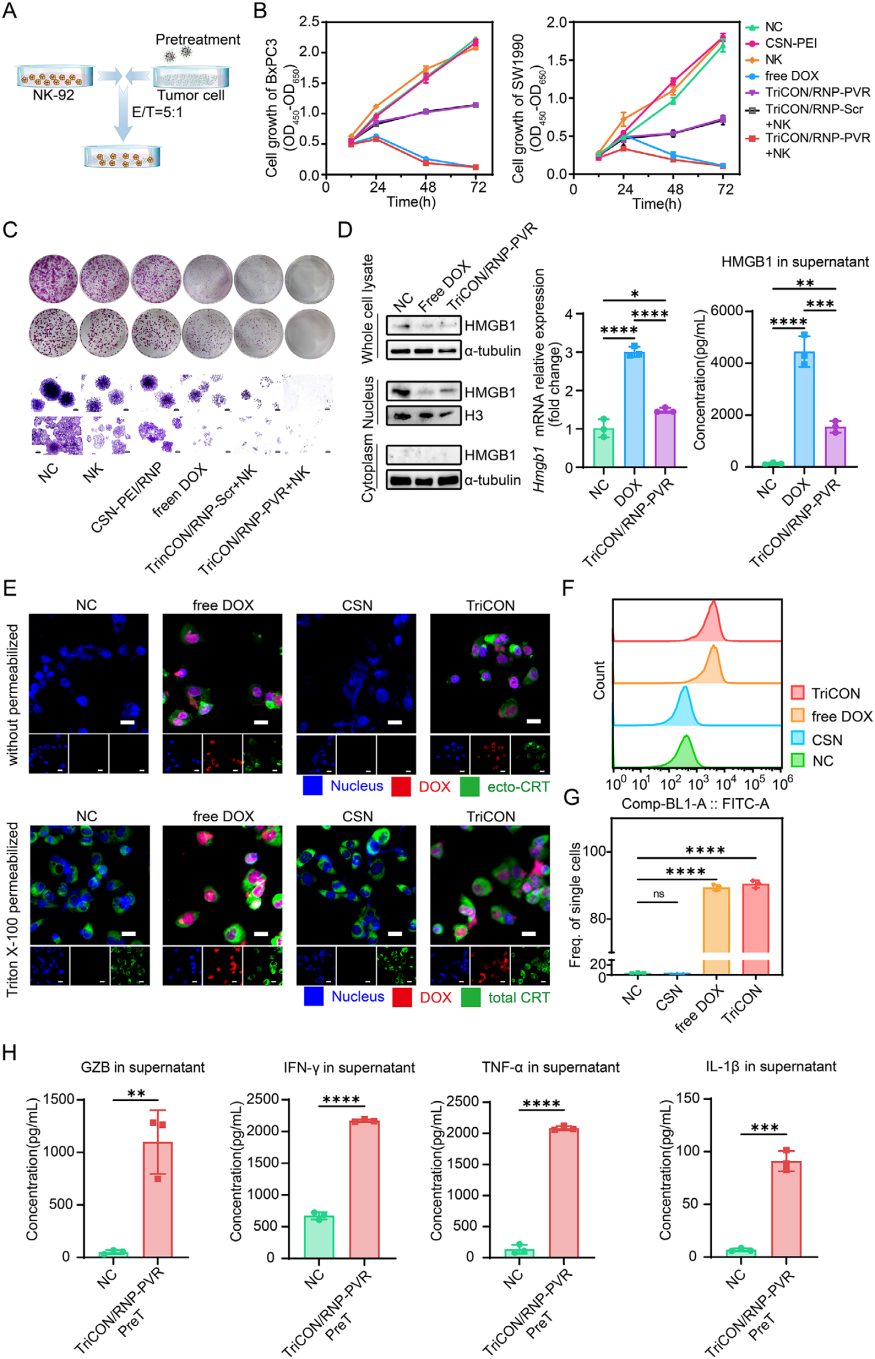
Activation of immunogenic cell death in vitro and enhanced NK cell killing ability. (A) Schematic diagram of co‐incubation of pretreated tumor cells and NK cells. (B) Growth and (C) clone formation capacity of tumor cells treated with CSN‐PEI, NK cells, free DOX, TriCON/RNP‐PVR, TriCON/RNP‐Scr + NK, TriCON/RNP‐PVR + NK cells. (D) Subcellular localization, expression, and extracellular secretion of HMGB1 in free DOX and TriCON/RNP‐PVR‐treated cells. (E) Representative immunofluorescence images of CRT exposed on the cell surface and total CRT following different treatments. Scale bar: 20 µm. (F) Representative flow cytometry and (G) corresponding quantitative analyses of ectropion of CRT protein after different treatments. (H) Secretion assay of GZB, IFN‐γ, TNF‐α, IL‐1β in tumor cells after DOX and TriCON/RNP‐PVR treatment. All data are presented as mean ± SD. Data in (B), (D), (G), and (H) are from *n* = 3 biologically independent samples. Statistical significance was assessed using one‐way analysis of variance (ANOVA). (ns for *p* > 0.05, ^*^ *p* < 0.05, ^**^ *p* < 0.01, ^***^ *p* < 0.001, ^****^ *p* < 0.0001).

PVR, as an immune checkpoint in cancer cells, plays a crucial role in mediating the activation of NK cells by binding to TIGIT, thereby acting as a ‘don't eat me’ signal. Given that the DNA‐damage response has been implicated in the upregulation of PVR in T cells through the promotion of reactive oxygen species (ROS) release [30], we firstly confirmed this finding in PDAC cells. As shown in Figure S7, our results showed that DOX significantly increased the expression of PVR in PDAC cell lines. We then try to identify that TriCON/RNP‐PVR enhanced the cytotoxic effect of DOX by synergistically silencing PVR. The cell growth was assessed using the CCK‐8 assay at indicated time points following the co‐culture of pre‐treated tumor cells and NK cells (Figure 4A). The data demonstrated that TriCON/RNP‐PVR + NK significantly suppressed cell growth compared to other groups, especially to the TriCON/RNP‐Scr + NK (Figure 4B). Additionally, the clone formation assay further confirmed the function of TriCON/RNP‐PVR (Figure 4C).

On the other hand, noting that DNA‐damaged tumor cells have been reported to enhance the cytotoxic activity of NK cells by releasing damage‐associated molecular patterns (DAMPs) to ICD of tumor cells. To identify whether the TriCON/RNP‐PVR induces ICD in PDAC, the levels of high‐mobility group box 1 protein (HMGB1) and intracellular adenosine triphosphate (ATP) were quantified following co‐incubation with DOX or TriCON/RNP‐PVR. As illustrated in Figure 4D, DOX significantly enhanced the expression of HMGB1, which thereby released from nucleus to the extracellular space. As indicated in Figure 4E,F, CRT immunofluorescence was significantly increased on membrane of dying SW1990 cells in TriCON and free DOX groups, compared with NC and CSN groups according to the corresponding quantification results of mean fluorescence intensity (MFI) (Figure 4G). Concurrently, the concentration of intracellular ATP was markedly increased (Figure S8), suggesting that DOX released by TriCON significantly triggered ICD in tumor cells. Given that DOX‐induced ICD is associated with robust cytokine production (Figure S9) [31], we then examined the cytokine response elicited by TriCON/RNP‐PVR using quantitative PCR (qPCR) and enzyme‐linked immunosorbent assay (ELISA). As demonstrated in Figure 4G, as compared to the untreated control or DOX‐treated group, TriCON/RNP‐PVR significantly elevated the expression of inflammatory cytokines, including CXCL10, IFN‐α1, IL‐1β, and TNF‐α in tumor cells, as well as the expression of antitumor effectors in the co‐culture system, such as GZB, IFN‐γ, IL‐1β, and TNF‐α. Collectively, these results suggest that DOX‐induced PDAC‐ICD activation holds significant potential for enhancing NK cell immune activation.

### 2.6 Tumor Growth Suppression of TriCON In Vivo

**FIGURE 5:**
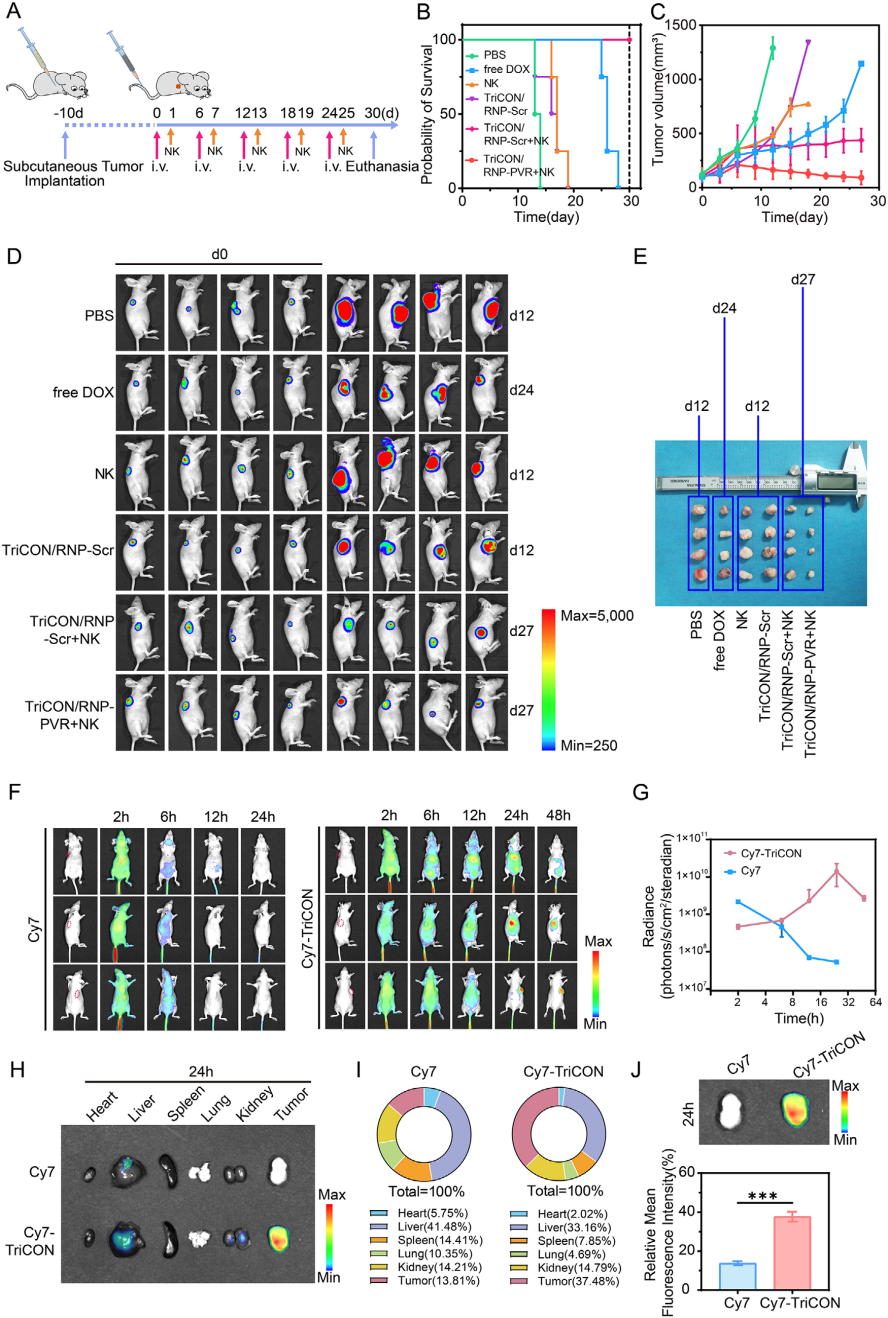
Antitumor efficacy of the TriCON/RNP‐PVR in vivo. (A) Administering the timeline and procedures of different formulations in SW1990 tumor‐bearing mice. (B) Survival curves for the mice that received the treatment of different formulations. (C) Tumor growth curves of the mice treated with PBS, free DOX, NK, TriCON/RNP‐Scr, TriCON/RNP‐Scr + NK, and TriCON/RNP‐PVR + NK. (D) Representative in vivo bioluminescence images of tumor regions in mice on Day 0 and at subsequent endpoints, specifically on Days 10, 12, 24, and 27 following the implantation of SW1990‐LUC cells. (E) Photographs of the excised tumors after treatment. (F) Representative in vivo fluorescence imaging and (G) quantitative assessment of fluorescence intensity of tumors at 2, 6, 12, 24, and 48 h following intravenous administration via the tail vein of Cy7 fluorescent dye and Cy7‐labeled TriCON nanoparticles. (H) Representative ex vivo fluorescence images and (I) quantitative measurements of fluorescence intensity in major mouse organs and (J) tumors obtained at 24 h postinjection. All data are presented as mean ± SD. *n* = 4 (except *n* = 3 in (H) and (J)). Statistical significance was assessed using two‐tailed Student's *t*‐tests. (^***^ *p* < 0.001).

To evaluate the antitumor effects of TriCON in vivo, the xenograft tumor model was conducted in BALB/c nude mice using SW1990‐LUC cells (Figure 5A). The TriCON was administered via the tail vein every 6 days, for a total of four times. Several groups were utilized to improve comparison, including PBS, free DOX, TriCON carrying sgScr (TriCON/RNP‐Scr), NK, TriCON carrying sgScr combined with NK cells (TriCON/RNP‐Scr + NK), and TriCON/RNP‐PVR combined with NK cells (TriCON/RNP‐PVR + NK). Then, the body weight and tumor volume were monitored and recorded throughout the 30‐day treatment period. As demonstrated in Figure S10, no significant weight loss was observed in the treatment groups as compared with the control. Following TriCON treatment, the histological pathology of tumor‐bearing mice showed no significant differences compared to the control group (Figure S11). In terms of biochemical parameters assessing liver and kidney function, renal impairment was observed in the free‐DOX group. However, no significant differences were noted between the other treatment groups and the saline control group (Figure S12), which suggesting that the TriCON/RNP‐PVR + NK is safe and suitable for potential clinical application in vivo. Importantly, we found the tumor growth was significantly suppressed in mice treated with TriCON/RNP‐PVR + NK and free DOX. In contrast, a rapid increase of tumor was observed in mice receiving PBS and NK cell injections. Impressively, TriCON/RNP‐PVR + NK treatment virtually eliminated tumors and significantly improved survival in mice (Figure 5D,E and Figure S13). To investigate the in vivo biodistribution of TriCON, Cy7‐labeled TriCON nanoparticles were intravenously injected through the tail vein in a mouse xenograft tumor model and subsequently monitored in real time utilizing an in vivo imaging system (IVIS). The findings revealed that tumors injected with Cy7‐TriCON nanoparticles exhibited fluorescence signal accumulation within 24 h postinjection, with the signal persisting up to 48 h. In contrast, the group receiving free Cy7 dye showed no fluorescence enrichment at the tumor site, and detectable signals dissipated within 24 h, likely due to metabolic clearance (Figure 5F,G,J, and Figure S14). Furthermore, the fluorescence intensity of Cy7‐TriCON nanoparticles in major organs of treated mice was comparable to that observed in untreated controls (Figure 5H,I), indicating minimal off‐target accumulation in normal tissues. In vivo fluorescence imaging combined with ex vivo imaging findings indicate that Cy7‐TriCON is predominantly metabolized through hepatic and renal routes within the organism.

**FIGURE 6:**
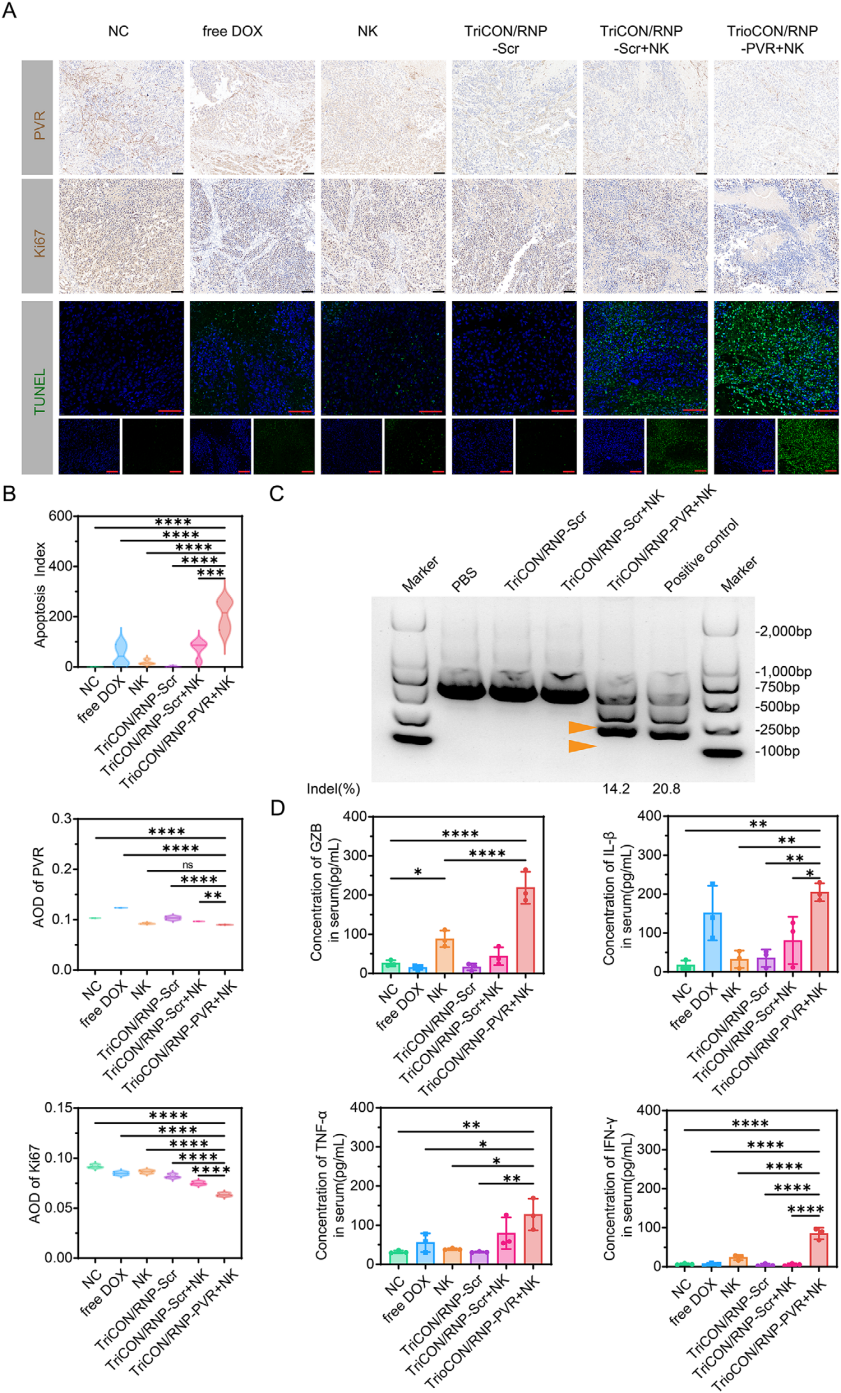
Antitumor efficacy of the TriCON/RNPPVR in vivo. (A) Immunohistochemical analysis of the tumor slices stained with (a) PVR and (b) Ki‐67. (c) Representative images of tumor sections stained with TUNEL. The scale bars are 100 µm. In TUNEL staining, apoptotic cell nuclei are stained green. (B) Quantification of Average Optical Density (AOD) of Immunohistochemical Images. (C) Indels of the PVR gene in tumor tissues treated with free RNP and TriCON/RNP‐PVR + NK treated, untreated. (D) GZB, IFN‐γ, TNF‐α, and IL‐1β levels in tumors from mice treated with different formulations. All data are presented as mean ± SD. Statistical significance was assessed using one‐way analysis of variance (ANOVA). (^*^ *p* < 0.05, ^**^ *p* < 0.01, ^***^ *p* < 0.001, ^****^ *p* < 0.0001).

Then, the terminal deoxynucleotidyl transferase dUTP nick end labeling (TUNEL) and Ki‐67 staining were performed to investigate the cell proliferation and apoptosis in tumors from each group. As indicated in Figure 6A,B, a significant decrease of Ki‐67 positive tumor cells (indicated in sepia) and an increase of TUNEL‐positive apoptotic tumor cells (indicated in green) were observed in the TriCON/RNP‐PVR + NK group compared to any of the other groups. Taken together, these findings suggest a powerful anti‐tumor capacity of the strategy TriCON/RNP‐PVR + NK in vivo.

In addition, we examined the expression level of PVR to clarify the genome editing efficacy of TriCON/RNP‐PVR in vivo, as evidenced by western blot and T7E1 assay. Clear cleavage bands were detected in TriCON/RNP‐PVR + NK‐treated mice, with indels of 14.2% (Figure 6C). Further assessment of in vivo gene editing efficiency was conducted using immunohistochemistry (IHC) on tumor tissues (Figure 6B). The TriCON/RNP‐PVR + NK groups exhibited lower PVR expression compared to both the TriCON/RNP‐Scr group and the TriCON/RNP‐Scr + NK group. We further examined the disruption of PVR in other organ tissues (e.g., heart, liver, kidney, spleen, brain, and lung) to exclude the off‐target editing of TriCON, as summarized in Figure S15, no significant disruption was observed in these organs. This finding is consistent with the in vitro results, suggesting that the gene disruption of PVR effectively contributes to the tumor suppression in vivo.

Furthermore, to illustrate the anti‐tumor immune response of NK cells elicited by the nanococktail strategy, we tested several cytokines secreted by NK cells. As demonstrated in Figure 6D, TriCON/RNP‐PVR + NK treatment significantly elevated the levels of TNF‐α, GZB, IL‐1β, and IFN‐γ in both tumor tissue and serum, as compared with TriCON/RNP‐Scr + NK group. These findings suggest that the nanococktail strategy has the potential to sensitize immunologically “cold” tumors for enhanced cancer immunotherapy.

### 2.7 Suppression of In Situ Pancreatic Cancer Growth by TriCON

**FIGURE 7:**
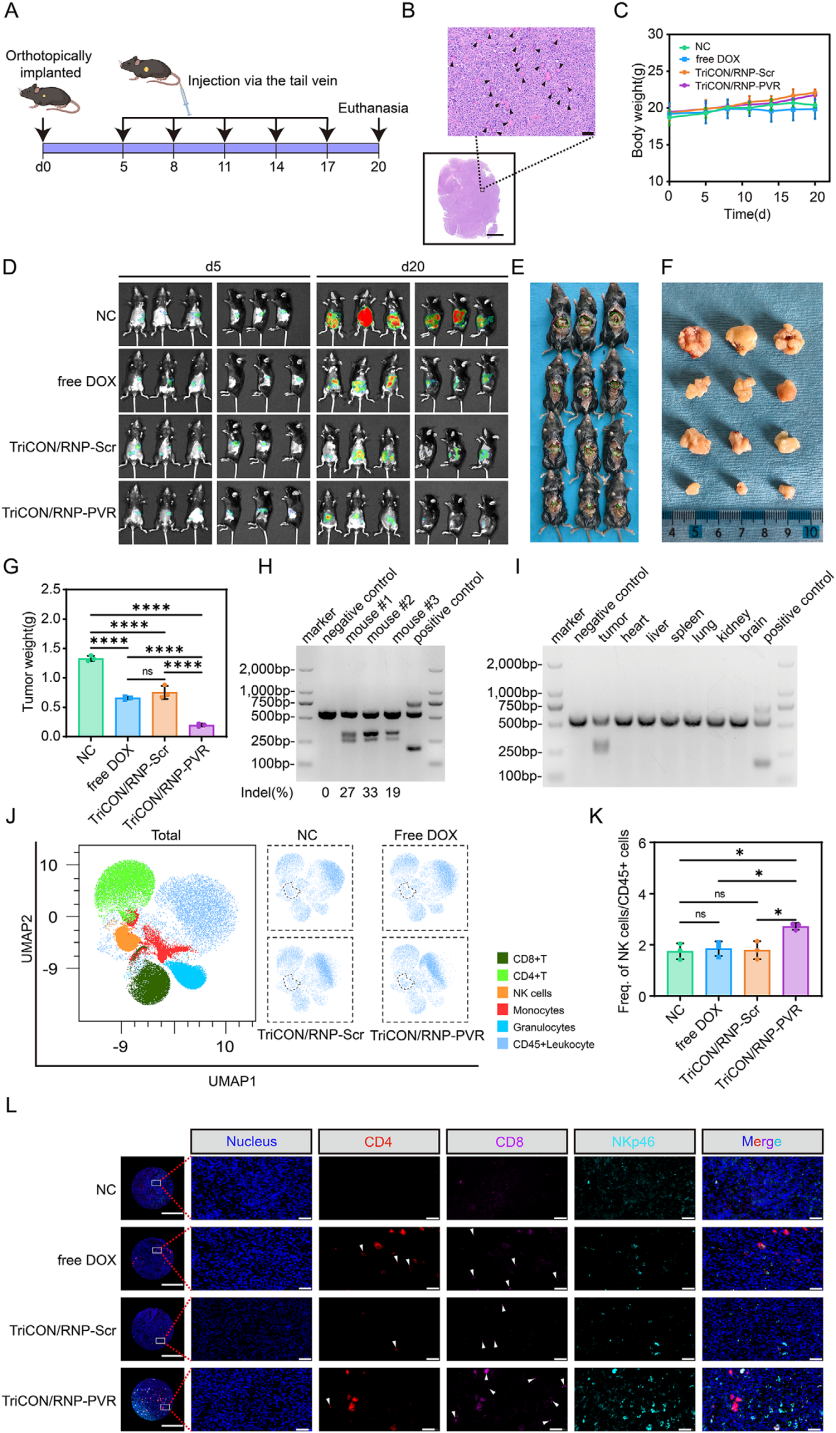
Evaluation of the anti‐tumor effectiveness of TriCON/RNPPVR in immunocompetent orthotopic xenograft models. (A) schematic representation of the timeline and procedural framework for administering various formulations within an in situ transplanted tumor model exhibiting immune activity. (B) HE‐stained section of in situ implanted tumor tissue. The lower inset shows the overall tissue field (scale bar = 2000 µm); the upper enlarged view depicts the area outlined by the dashed box, with black triangles indicating pancreatic acinar tissue (scale bar = 50 µm). (C) Changes in body weight after treating the mice with different formulations. (D) Representative in vivo bioluminescence images of tumor regions in mice on Day 5 and Day 20 following the implantation of Pan02‐LUC cells. (E) Representative images of mice euthanized and fixed in the supine position. Tumor tissues, indicated by green circles, are distinctly observable within the abdominal cavity. (F) Photographs of the excised tumors after treatment. (G) Indels of the PVR gene in tumor tissues treated with TriCON/RNP‐PVR. (H) Indels of the PVR gene in several tissues of mice treated with TriCON/RNP‐PVR. (I) Weight of the excised tumors after treatment. (J) The UMAP visualization illustrated the distribution of immune cell populations across various treatment conditions. The overview panel provided an integrated representation of all samples, whereas the subsequent four panels depicted UMAP projections corresponding to each individual treatment group. The cluster identified as NK cells was delineated by a dashed ellipse. (K) The ratio of NK cells to CD45‐positive cells within each treatment group. (L) Multiplex immunofluorescence staining analysis of immune cells in tumor tissue. The left panel shows an overview scan image of a representative tumor tissue section (scale bar = 500 µm). The enlarged view of the boxed area demonstrates details of immune cell infiltration (scale bar = 50 µm). The white triangles indicate single‐positive cells. All data are presented as mean ± SD. *n* = 3. Statistical significance was assessed using one‐way analysis of variance (ANOVA). (^*^ *p* < 0.05, ^**^ *p* < 0.01, ^***^ *p* < 0.001, ^****^ *p* < 0.0001).

To further evaluate the activating effect of TriCON on NK cells, an orthotopic tumor model was performed using Pan02‐LUC cells in C57BL/6 mice (Figure 7A). Following in situ implantation of tumor cells, we administered TriCON/RNP‐PVR treatment via tail vein injection at specific time points (days 5, 8, 11, 14, and 17). As shown in Figure 7B, the HE staining results clearly reveal the typical histopathological features of in situ pancreatic cancer. The body weights remained stable in all experimental groups, with no statistically significant difference observed between groups (Figure 7C). As shown in Figure 7D–F, compared with the control group, the TriCON/RNP‐PVR treatment group exhibited a significant reduction in tumor size, indicating a potent antitumor effect. Following the conclusion of treatment, we excised and weighed the pancreatic tumors from mice in each group. This result was confirmed by tumor weight (Figure 7G).

To confirm the therapeutic efficacy of TriCON/RNP‐PVR from its specific gene editing function, we analyzed the editing efficiency of the PVR gene using the T7E1 assay. Genomic analysis of tumor tissue (Figure 7H) revealed efficient PVR gene editing bands detected in the TriCON/RNP‐PVR treatment group, demonstrating that this therapy successfully achieved the expected genomic alterations at the tumor site. Immunohistochemical staining results (Figure S16) also confirmed the attenuation of PVR expression by TriCON/RNP‐PVR. Meanwhile, to ensure treatment safety, we conducted off‐target effect assessments on the genomes of organs, including the heart, liver, spleen, lungs, kidneys, and brain. T7E1 detection results (Figure 7I) indicate that no editing signals for the PVR gene were detected in these organs. This fully demonstrates that TriCON exhibits high tumor targeting in vivo without inducing detectable systemic off‐target editing, further supporting its favorable safety profile.

To investigate the immunological mechanism by which TriCON/RNP‐PVR inhibits tumor growth, we analyzed NK cell infiltration and activation within the tumor microenvironment. Quantification of tumor‐infiltrating lymphocytes via flow cytometry revealed (Figure 7J,K, and Figure S17) that NK cell infiltration levels in tumor tissues were significantly higher in the TriCON/RNP‐PVR treatment group compared to other groups. Additionally, we further assessed the activation status of NK cells through multiplex immunofluorescence staining. As shown in Figure 7L, NK cells within tumors in the TriCON/RNP‐PVR treatment group exhibited stronger signaling of activation markers. These results were further validated by qPCR (Figure S18) and ELISA (Figure S19). Collectively, these data indicate that targeted editing of the PVR gene not only enhances the recruitment of NK cells to tumor sites but, more importantly, effectively activates the cytotoxic functions, thereby synergistically eliciting potent antitumor immune responses.

## 3 Conclusions

In summary, we developed a triple‐modality therapeutic platform, TriCON, designed for targeting intracellular delivery of RNP and disruption of the immune checkpoint PVR gene in pancreatic cancer. This nanococktail is readily synthesized, demonstrates high protein loading efficiency, and retains the activity of functional proteins when mixed with protein solutions. The incorporation of targeting peptides on the nanovehicle surface enhances endocytosis. Our findings indicate that this nanococktail can significantly inhibit tumor growth and promote NK cell activation and killing in vitro. Notably, the delivery of Cas9 protein and sgRNA via these nanovectors achieves high gene editing efficacy in PVR within tumor‐bearing mice, effectively suppressing tumor growth. These results underscore the potential of CSN nanovectors for gene editing system delivery, enhancing immunotherapy outcomes in pancreatic cancer through co‐injection with NK cells, and providing valuable insights for future clinical applications.

## 4 Experimental Section

The details about the synthesis, characterizations, cell preparation, biological assay, and analysis are in the Supplementary data.

## Author Contributions

X.P., F.W., Z.Q. and Y.L. conceived the concept and experimental design; X.P., J.H., S.L., J.Z. and H.L. performed the research; X.P., J.H., S.L., L.Z. and H.L. analyzed the data; X.P., F.W., Z.Q. and Y.L. wrote the paper. All authors have read and approved the final version of the manuscript.

## Conflicts of Interest

The authors declare no conflicts of interest.

## Supporting information




**Supporting File**: advs73856‐sup‐0001‐SuppMat.docx.

## Data Availability

The data that support the findings of this study are available from the corresponding author upon reasonable request.
