# Supplementary material for: TriCON: A Carbon‐Based Triple‐Modal Nanoplatform for Pancreatic Cancer Therapy
Source: Adv Sci (Weinh). 2026 Jan 18;13(17):e12978. doi: 10.1002/advs.202512978 (PMC13042355; doi:10.1002/advs.202512978)
Supplement: Supplementary file 1 — Supporting File: advs73856‐sup‐0001‐SuppMat.docx. [file ADVS-13-e12978-s001.docx]

***Supporting Information***

**TriCON: A Carbon-Based Triple-Modal Nanoplatform for Pancreatic Cancer Therapy**

*Xinyu Peng ^a1^, Jiaxing Huang ^b^, Shengnan Lv ^a^, Jian Zhang ^a^, Liangliang Zhang ^b^, Huan Liu ^a^, Yan Liu ^c,d^*, Zhen-An Qiao ^b^*, Feng Wei ^a^*, and Bao-Lian Su ^e,f^*

X. Peng, S. Lv, J. Zhang, H. Liu, Prof. F. Wei

Jilin University

1. Hepatobiliary Pancreas Department, General Surgery Centre, The First Hospital of Jilin University, Xinmin Street 1, Changchun 130021, P. R. China

*Corresponding Author: wei_feng@jlu.edu.cn

J. Huang, L. Zhang, Prof. Z.-A. Qiao

Jilin University

1. State Key Laboratory of Inorganic Synthesis and Preparative Chemistry, College of Chemistry, Qianjin Street 2699, Changchun 130012, P. R. China

*Corresponding Author: qiaozhenan@jlu.edu.cn

Prof. Y. Liu

1. Key Laboratory of Jilin Province for Zoonosis Prevention and Control, Institute of Military Veterinary Medicine, Academy of Military Medical Sciences, Liuying West Road 666, Changchun 130117, P. R. China
2. State Key Laboratory of Pathogen and Biosecurity, Changchun Veterinary Research Institute, Chinese Academy of Agricultural Sciences, Liuying West Road 666, Changchun 130117, P. R. China

*Corresponding Author: liu820512@163.com

Prof. B.-L. Su

1. State Key Laboratory of Advanced Technology for Materials Synthesis and Processing, Wuhan University of Technology, 122, Luoshi Road, Wuhan 430070, China
2. Laboratory of Inorganic Materials Chemistry (CMI), University of Namur, 61 rue de Bruxelles, B-5000 Namur, Belgium

E-mail: bao-lian.su@unamur.be

**Experimental Section**

1. Ethical issues

All animal experiments were performed according to protocols in accordance with policies of the National Ministry of Health and approved by the Laboratory Animal Center of the First Hospital of Jilin University (11970). The maximum permissible tumor volume is 1,500 cubic millimeters, and the maximum allowable tumor diameter does not exceed 2 centimeters (Guidelines for Endpoints in Animal Research Protocols).

1. Chemicals and reagents

All the chemical reagents and solvents not specifically noted were purchased from Sigma-Aldrich (Shanghai, China). Dialysis membranes 3.5/50 kDa (#F132590, #F131372) and HMGB1 ELISA kit (#D711210) were obtained from Sangon Biotech (Shanghai, China). Cas9 nuclease (#Z03385) was from GenScript (Nanjing, China). Dulbecco’s Modified Eagle’s Medium (DMEM, #10-017-CV), Phosphate Buffered Saline (PBS, #21-040-CV), RPMI 1640 (#10-043-CV), and DMEM/F12 50:50 Mix (#15-090-CV) were supplied by Corning (NY 14831, USA). Fetal bovine serum (FBS, #A5669701), trypsin (#25200072), and penicillin/streptomycin (#15070063) were from Gibco (Thermo Fisher, USA). The Granzyme B ELISA kit (#BSM2027-2, #BMS6029), IFN-γ ELISA kit (#KHC4021, #KMC4021), TNF-α ELISA kit (#BMS2034, #BMS607-3), perforin ELISA kit (#BMS2306TEN) and Alexa Fluor 594 NHS Esters (#A20104) were from Invitrogen (Thermo Fisher, USA). The IL-1β ELISA kit (#97028ES48, #98024ES48) was from Yeasen (Shanghai, China). FITC (#P0639S), paraformaldehyde (#P0099), Bicinchoninic acid (BCA) protein assay kit (#P0009), Hoechst 33342 (#C1025), lactate dehydrogenase (LDH) cytotoxicity assay kit (#C0017), and 4,6-diamidino-2-phenylindole dihydrochloride (DAPI, #C1006) were purchased from Beyotime Biotechnology (Shanghai, China). The cell-penetrating peptides were purchased from MedChemExpress (MCE, #HY-P5307). The amino acid sequence of CPP is as follows:

Gly-Leu-Phe-Glu-Lys-Ile-Glu-Gly-Phe-Ile-Glu-Asn-Gly-Trp-Glu-Gly-Met-Ile-Asp-Gly-Trp-Tyr-Gly-Tyr-Gly-Arg-Lys-Lys-Arg-Arg-Gln-Arg-Arg. Cy7-NHS ester (#S53893) were obtained from Shanghai Yuanye Bio-Technology (Shanghai, China). T7 Endonuclease I (#E3321) was acquired from New England Biolabs (Beijing, China). Dimethyl sulfoxide (DMSO, # D8371) was obtained from Solarbio Science & Technology (Beijing, China). The cell genomic DNA extraction kit (#DP304) and DNA purification kit (#DP204) were purchased from TIANGEN Biotech (Beijing, China). Cell Counting Kit-8 (CCK-8, #CK04) was obtained from Dojindo Laboratories (Shanghai, China). Fluorescent TUNEL staining kit (#G1501) was obtained from Servicebio (Wuhan, China). Apparent pKa Assay Kit (#702680) were purchased from Cayman Chemical (Michigan, USA) All details about the antibodies are shown in Table S1.

1. Synthesis of PEI-coated Mesoporous Carbon Hollow Spheres

Hollow mesoporous carbon spheres were synthesized using a one-pot, surfactant-free approach similar to the previous study by the Yu Lab.[1] In short, a 1:5 molar ratio of tetrapropyl orthosilicate (TPOS, Sigma #679240)/tetraethyl orthosilicate (TEOS, Sigma #131903) mixture (with a total silicon amount of 12 mmol) was added to a solution containing 27 mL of ethanol (Sigma #[493511](https://www.sigmaaldrich.cn/CN/zh/product/sial/493511)), 13 mL of H_2_O, and 1.5 mL of NH3·H_2_O (25 wt. %, Sigma #17093). After vigorously stirred at room temperature for 15 minutes, resorcinol (0.22 g, Sigma #398047) and formaldehyde (0.308 mL, 37 wt. %, Sigma #F8775) were added to the above solution and stirred for 36 hours. The precipitates were separated by centrifugation, washed with deionized water and ethanol several times, and dried at 50 °C overnight. Then, the mesoporous hollow carbon spheres were obtained after carbonization at 700 °C under N_2_ for 5 hours and removal of silica by hydrofluoric acid (HF, 10 wt. %, Sigma #1.00335) for 24 h at room temperature, followed by neutralization and dialysis against deionized water for 72 h.

For the carboxylation of CSN, CSN nanoparticles (0.5 g) were dispersed in a solution containing ammonium persulfate (APS, 30 mL, 0.25 mol/L, Sigma #1.01200) and H_2_SO_4_ (1.6 mL, 18.4 mol/L, Sigma #1.00732). The mixture was stirred at 60 °C with reflux in a 100 mL round-bottom flask for 3 hours. The carboxylated CSN was harvested by centrifugation, purified repeatedly with water and ethanol until reaching a neutral pH, then dried under vacuum to obtain CSN-COOH.

N-hydroxystearimide (NHS, 19 mg, Sigma #130672) and 1-(3-dimethylaminopropyl)-3-ethylcarbodiimide (EDC, 32 mg, Sigma #39391) were added to a mixture containing N, N-dimethylformamide (DMF, 30 mL, Sigma #494488) and CSN-COOH (30 mg). The mixture was reacted for 30 minutes at ambient temperature to activate the carboxyl groups. Subsequently, excess Branch polyethyleneimine (PEI, Mw ~25,000, Sigma #408727) was added and stirred for 5 hours. The obtained CSN-PEI was separated by centrifugation and washed three times with distilled water to remove the DMF buffer and any unreacted PEI.

1. Design and synthesis of sgRNA and primers

The gRNAs were designed by the Synthego website (https://design.synthego.com/). Suitable primers were designed from NCBI BLAST. These results were shown in Figure S20. The synthesis and quality inspection of gRNAs and primers were completed by Suzhou GenePharma Co., Ltd and Jilin Comate Bioscience Co., Ltd.

1. Synthesis and purification of TriCON/RNP nanoparticles

Cas9 protein and sgRNA (1:2, based on molar quantities) were initially mixed in PBS for 15 minutes to form CRISPR-Cas9 ribonucleoprotein (RNP). The RNP was then mixed with the prepared TriCON nanoparticles at 4 °C overnight. After centrifugation, cell-penetrating peptide (CPP) was encapsulated on the outside of TriCON/RNP. The weight ratio of CPP to TriCON was 5:1 (based on the mass) and further equilibrated for 5 minutes at room temperature. Additionally, RNP was ligated with Alexa Fluor 594-NHS to monitor the internalization process of TriCON/RNP.

1. Nanoparticle characterization

1）Size and zeta potential of nanoparticles

The hydrated particle sizes and zeta potentials of CSN, CSN-COOH, CSN-PEI, and TriCON were measured by dynamic light scattering instrument (Malvern Nano ZS90).

1. Scanning electron microscopy (SEM) and transmission electron microscopy (TEM) morphological characterization

The morphology was obtained by a JEOL JSM 6700F Scanning Electron Microscopy (SEM). Transmission electron microscopy (TEM) was performed on a Philips-FEI Tecnai G2S-Twin microscope (200 kV field emission gun).

3) X-ray photoelectron spectroscopy (XPS)

XPS curves were obtained on a ESCALAB250 Al Kα radiation instrument to determine the compositions and the valence states of the elements in the samples.

4) Nitrogen adsorption/desorption determination

Nitrogen adsorption-desorption measurements were performed using a Micromeritics ASAP 2420 4200e nitrogen adsorption analyzer.

5) Fourier transform infrared spectroscopy (FTIR)

Fourier transform infrared spectroscopy analysis was performed using a Perkin-Elmer 580B infrared spectrometer.

1. Encapsulation and loading efficiency of DOX in vitro

As in a previous study, we investigated the loading, encapsulation, and release rates of DOX in vitro.[2] Briefly, 20 mg of DOX was dissolved in 5 mL of water, and then 5 mg of CSN-COOH was dispersed in the solution. The mixture was ultrasonicated for 30 minutes and stirred in dark at room temperature for 12 hours. Followingly, 5 mL of PBS at pH 7.4 was added and stirred for 12 hours. The DOX-loaded CSN-COOH (DOX@CSN-COOH) was collected by centrifugation and washed several times with PBS to remove the surface-unencapsulated DOX. The amount of DOX was measured using a UV spectrophotometer (Infinite M1000 Pro, Tecan, Switzerland) by collecting the supernatant from each centrifugation step. The quantity of encapsulated DOX was calculated by subtracting the remaining DOX concentration in the supernatant after loading and washing from the initial DOX concentration. The drug loading and encapsulation efficiencies were calculated using the following equations:

Loading efficiency (%)$=100\times\frac{total DOX added - DOX in supernatant}{total nanocarriers added + DOX loaded in the nanocarriers}$

Encapsulation efficiency (%) $=100\times\frac{total DOX added - DOX in supernatant}{total DOX added}$

1. In vitro release of DOX

The 1 mg sample of DOX-loaded nanoparticles (DOX@CSN-COOH) were dispersed in 5 mL of PBS with different pH values (pH 5.5, pH 6.5, and pH 7.4) and incubated at 37 °C on a shaker. Subsequently, 1 mL of the released solution was withdrawn from the suspension at predetermined time intervals and replaced with an equal volume of fresh PBS. The percentage release of DOX from each group was measured using UV spectrophotometry at an excitation wavelength of 484 nm.

1. Gel retardation assay

To investigate the ability of CSN to form complexes with RNP, an electrophoretic mobility shift assay was performed. Briefly, the CSN-PEI solution was mixed with an equal volume of RNP (1 μg) at mass ratios ranging from 0:1 to 30:1. The mixture in each tube was thoroughly vortexed and then incubated for 30 minutes at room temperature. The RNP bands were electrophoresed on 10% SDS-PAGE gels with Tris-Glycine buffer at 110 V for one hour. Subsequently, the gels were stained with Coomassie Brilliant Blue, and the RNP bands were visualized using a gel documentation analysis system (Tanon 5200, Tanneng, Shanghai).

1. Evaluation of Cas9 RNP release in vitro

100 μg of TriCON/RNP nanoparticles were added to 1 mL of PBS (pH 7.4, pH6.5 and pH 5.5) and incubated at 37 °C. 100 μL of the solution was collected at indicated time points of incubation (0, 4, 8, 12, 16, 24, 36, 48, and 72 h), the protein concentration in the supernatant was determined by using a BCA kit. The release of RNP was quantified by calculating the ratio of the cumulative amount of RNP released to the initially loaded amount of RNP.

1. Stability of nanoparticles in vitro

The TriCON was added to PBS or 1640 medium containing 10% FBS for different incubation time. Samples were collected at the predesigned time intervals to monitor the variations in particle size and PDI index (Table S2).

1. Cell culture

All the used cell lines HPDE6C7 (RRID: CVCL_0P38), SW1990 (RRID: CVCL_1723), PANC1 (RRID: CVCL_0480), BxPC3 (RRID: CVCL_0186) and NK92 (RRID: CVCL_2142) were obtained from American Type Culture Collection (ATCC) and maintained in our laboratory. HPDE6C7 and BXPC3 cells were cultured in 1640 medium, PANC1 cells in DMEM medium, and SW1990 cells in DMEM/F12 medium, each medium containing 10% (v/v) FBS and 100 units/mL of penicillin and streptomycin. NK92 cells were cultured in NK92-specific medium containing 12.5% FBS, 12.5% horse serum, and additional supplements including inositol, folic acid, L-glutamine, sodium bicarbonate, and 200 units of interleukin-2 per milliliter. All cells were cultured in a humidified environment with 5% CO_2_ at 37 °C. All cells were assayed using Short Tandem Repeats (STR) consistent with ATCC standards and confirmed to be free of Mycoplasma infection.

1. Cell viability assay

CCK-8 cytotoxicity assay was performed to examine the effects of CSN-based drugs on cell viability. In short, 0.8-1×10^4^ cells per well were seeded into a 96-well plate. After culturing overnight, the medium was replaced with fresh medium containing different concentrations of nanomedicine and incubated at 37 ℃ for further 72 hours. At predetermined time points, the medium was completely replaced with a solution containing 10% CCK-8. The absorbance of each well was measured at 450 nm. Cell viability was quantified as follows:

Cell viability (%) = $100\times\frac{\mathrm{OD}\mathrm{sample}-OD\mathrm{blank}}{\mathrm{OD}\mathrm{control}-OD\mathrm{blank}}$

The OD_sample_ and OD_control_ represent the absorbance values of treated and untreated control cells, respectively. OD_blank_ refers to the absorbance of wells containing unplanted cells. Six independent experiments were carried out in all experiments.

1. Lactate dehydrogenase (LDH) release assay

BxPC3 or SW1990 cells were seeded in 96-well plates at a density of 1×10^4^ cells per well. After incubating overnight at 37 °C, the medium containing the drug was replaced with fresh medium and incubated for 12 hours. Subsequently, all the media were discarded and replaced with NK92 culture at a density of 5×10^4^ cells per well. The cell supernatant was collected at the designated time points for detection. An LDH cytotoxicity assay kit was utilized to measure the level of LDH in the supernatant. The data were standardized against cells co-cultured with complete medium only (negative control) or with medium containing 1% Triton X-100 at 37 °C for 30 minutes (positive control).

1. RNA extraction and quantitative real-time reverse transcription polymerase chain reaction(qRT--PCR)

Total RNA was isolated using an extraction kit (BSC52, BioFlux, China). An equivalent amount of total RNA was used as a template for complementary DNA (cDNA) synthesis. The target genome copies were quantified through real-time quantitative PCR using SYBR Green PCR Master Mix (RR820, TAKARA, China), following the manufacturer's instructions. The primer sequences for the target genes are provided in Table S3. The GAPDH transcript served as an internal reference, and the average value of each gene duplication was used to calculate the normalized relative abundance, expressed as 2^-ΔΔCT^.

1. Western blot assay

Cells were lysed with RIPA buffer containing Halt Protease & Phosphatase Inhibitor Cocktail on ice (Thermo 78442). The obtained protein was quantified using a BCA protein assay kit (Beyotime P0010). Twenty micrograms of protein samples were separated by SDS-PAGE and subsequently transferred onto polyvinylidene fluoride (PVDF) membranes (Merck ISEQ00010). Following a 40-minute blocking step with 5% skimmed milk at room temperature, the samples were incubated overnight with a primary antibody. Afterward, the blots were incubated with an HRP-conjugated secondary antibody, and visualization was performed using the Omni-ECL Femto Light Chemiluminescence Kit (EpiZyme SQ201) and the Tanon-5200 system.

1. Quantification of cytokines

To evaluate cytokine secretion with CSN/RNP treatment, BxPC3 cells were seeded in a 24-well plate at a density of 1×10^5^ cells per well and incubated overnight. Then, five times the number of NK92 cells were added and incubated together for 24 hours. The supernatant was collected through centrifugation, and the secretion levels of IFN-γ, TNF-α, granzyme B, IL-1β, and perforin were measured by ELISA kit, following the manufacturer's instructions.

1. pKa determination

The pKa value of TriCON was determined using the fluorescent probe 2-(p-toluidino)-6-napthalene sulfonic acid (TNS). First, we prepare a 1 mg/mL TriCON liquid in PBS solution. Then, prepare a series of TriCON solution with pH ranged from 3.00 to 10.00 containing 1 μM TNS, 10 mM HEPES, 10 mM 4-morpholineethanesulfonic acid, 10 mM ammonium acetate and 130 mM NaCl. The fluorescence intensity of each solution was recorded by a spectrophotometer with an excitation and emission wavelengths of 321 nm and 445 nm, respectively. The fluorescence data was analyzed using the sigmoidal best fit analysis. The pKa value was defined as the pH giving rise to the half-maximal fluorescence intensity (Figure S21).

1. Cleavage assay and quantification of gene editing efficiency

To quantify the editing efficacy of RNP in the target sequence, the genomic DNA was extracted using the Tianamp Genomic DNA Kit (Tiangen Biotech 4992254). The 819/616 bp sequence flanking the cleavage sites was amplified by PCR, and the PCR products were purified using a PCR purification kit (Tiangen Biotech 4992197). After annealing, the destruction efficiency was assessed using the T7 Endonuclease I Kit. The digested DNA products were separated by agarose gel electrophoresis and visualized under ultraviolet light. ImageJ software (Version 1.53c) was employed to quantify the relative intensities of each band. The indel of the target gene was quantified using the formula: % gene modification = 100 × (1 - (1 - fraction cleaved)^1/2^) as previously reported.[3] In this formula, the fraction cleaved is defined as the ratio of the sum of the cleavage products to the total intensity, which includes both the cleavage products and the undigested PCR matrix. It was assumed that the combined intensity of the three bands (a target sequence and two cleavage products) in the same lane corresponded to 100% of the DNA signal. Additionally, no cleaved bands were observed in the negative control. The PCR product was used for Sanger sequencing. The data from Sanger sequencing were analyzed using the Synthego ICE Analysis Tool (https://www.synthego.com/) and quantified for small indels.

1. Assessment of off-target effects

Cas-OFFinder (http://www.rgenome.net/cas-offinder/) was used to predict potential off-target sites. BxPC3 cells were transfected with CSN/PVRRNP and cultured for 48 hours. Genomic DNA was extracted, and two potential off-target sites were amplified by PCR. The PCR products were detected with the T7E1 mutation detection kit (refer to Table S3 for off-target sequences and primer sequences). These results were shown in Figure S22.

1. Immunofluorescence

The cells were incubated with 100 μg/mL CSN/RNP (sgRNA labeled with Alexa Fluor 594 dye) for 1, 2, 4, 6, and 12 hours. Then, the medium was removed, and the cells were washed with PBS. The cells were fixed with 4% paraformaldehyde (PFA) at room temperature for 20 minutes, followed by washing with PBS and permeabilization with 0.25% Triton X-100 for 5 minutes. Endosomes were labeled with 10 μg/mL anti-EEA-1 antibody, and lysosomes were labeled using LAMP1, followed by a green fluorescent-conjugated secondary antibody. Finally, the nucleus was stained with Hoechst 33342 and the staining were observed and photoed by using fluorescence microscopy (IX73P2F, IXplore Standard, Olympus, Japan).

1. Detection of immunogenic cell death (ICD) in vitro

SW1990 cells (2 × 10⁵ cells per well) were typically seeded into 6-well plates and allowed to incubate overnight. Subsequently, the cells were incubated with PBS, free DOX, and TriCON for 6 hours, respectively. Intracellular and extracellular levels of HMGB1 were examined using Western Blot and ELISA. Additionally, the release of ATP was quantified through a luciferase assay. All procedures were conducted according to the manufacturer's instructions. Additionally, CRT membrane exposure detection was performed using immunofluorescence and flow cytometry.

1. Synergistic antitumor efficacy in vivo

Female BALB/c immunodeficient nude mice (RRID: IMSR_RJ: BALB-C-NUDE，18-20 g) were purchased from Beijing Vital River Laboratory Animal Technology Co., Ltd. (Beijing, China). The mice were housed on a 12-hour light/12-hour dark cycle in a pathogen-free environment. All animal procedures were approved by the Animal Ethics Committee of the First Hospital of Jilin University. The mice were injected subcutaneously with 200 μL of SW1990-LUC cell suspension containing 1 × 10^7^ cells in sterile PBS buffer. When the tumor size grew to approximately 50 mm³, the mice were divided into six groups (n = 4): (1) NC; (2) free DOX; (3) NK; (4) TriCON/RNP-Scr; (5) TriCON/RNP-Scr+NK; and (6) TriCON/RNP-PVR+NK. The tumor-bearing mice in each group were intravenously injected with 0.45 mg/mL DOX equivalents of the respective drugs (150 μL per mouse), while the NC group was treated with an equal volume of sterile PBS. Tumor volume and body weight in each group were monitored every three days. The volume (V) of the tumor was calculated by the following equation: V = A × B^2^ / 2, where A and B represent the longer and shorter diameters of the tumors, respectively.

1. Histological examination and long-term toxicity assessment

Tumor-bearing mice were sacrificed on day 30. The serum samples were collected for the test of alanine aminotransferase (ALT), alkaline phosphatase (ALP), blood urea nitrogen (BUN) and creatinine (CRE). The major organs (tumor, heart, liver, spleen, lung, brain, and kidney) were collected, fixed in 4% PFA, and embedded in paraffin. Tumor sections were prepared and stained with hematoxylin and eosin (H&E). Additionally, TUNEL staining, as well as Ki67 and PVR immunohistochemical staining was performed according to the previously reports [4]. All microscopic images were acquired using a slice scanner (Pannoramic MIDI, 3DHISTECH, Hungary), and image processing was performed with CaseViewer 2.3.

1. In vivo biodistribution

To develop a pancreatic cancer model, SW1990-LUC cells (1×10⁶ cells per mouse) were subcutaneously implanted into the right dorsal region of Balb/C nude mice. Upon tumor volumes reaching approximately 200 mm³, a 100 μL dose of Cy7-labeled TriCON nanoparticles (20 μg) was administered via tail vein injection. Fluorescent imaging was performed using the Lago X system (Spectral Instruments Imaging, USA) at 2, 6-, 12-, 24-, and 48-hours following injection. At 24 hours post-injection, tumors as well as heart, liver, spleen, lung, and kidney tissues were harvested and subjected to imaging with the IVIS imaging system.

1. Anti-tumor effect in orthotopic models

An orthotopic pancreatic cancer model was developed in C57BL/6 mice through the surgical implantation of Pan02-LUC cells into the pancreas at a concentration of 1 × 10⁶ cells per mouse. Five days following surgery, the mice were randomly assigned to one of four treatment groups. Each group received intravenous tail vein injections of 100 μL of either PBS, free DOX, TriCON/RNP-Scr nanoparticles, or TriCON/RNP-PVR nanoparticles, with the latter two formulations containing 10 μg of Cas9 per mouse. Injections were administered every three days, and body weights were monitored throughout the treatment period. Three days after the final injection, at which point the tumor volume in the control group was approximately 1500 mm³, the mice were euthanized following blood collection. Tumor tissues were subsequently excised for weight measurement, histological examination, and immunological analyses. Additionally, spleens were harvested for flow cytometric evaluation.

1. Flow cytometry

Resuspend cells to a density of 1 × 10⁶ cells/mL. Add 4 μL of staining solution containing CD45, CD3, CD4, CD8a, NK1.1, Ly6G, LIN, and CXCR3 to 300 μL of cell suspension. Incubate at room temperature for 30 minutes, then analyze using a flow cytometer (BD, San Jose, CA, USA). Three biological replicates were performed per experiment.

1. Statistical Analysis.

One-way analysis of variance (ANOVA) or unpaired two-tailed student's t-test in the software GraphPad Prism (version 9.5) were used for statistical analysis and plotting, * p＜0.05，** p＜0.01，*** p＜0.001，**** p＜0.0001. All data have been repeated at least three times, and finally shown as mean ± SD.


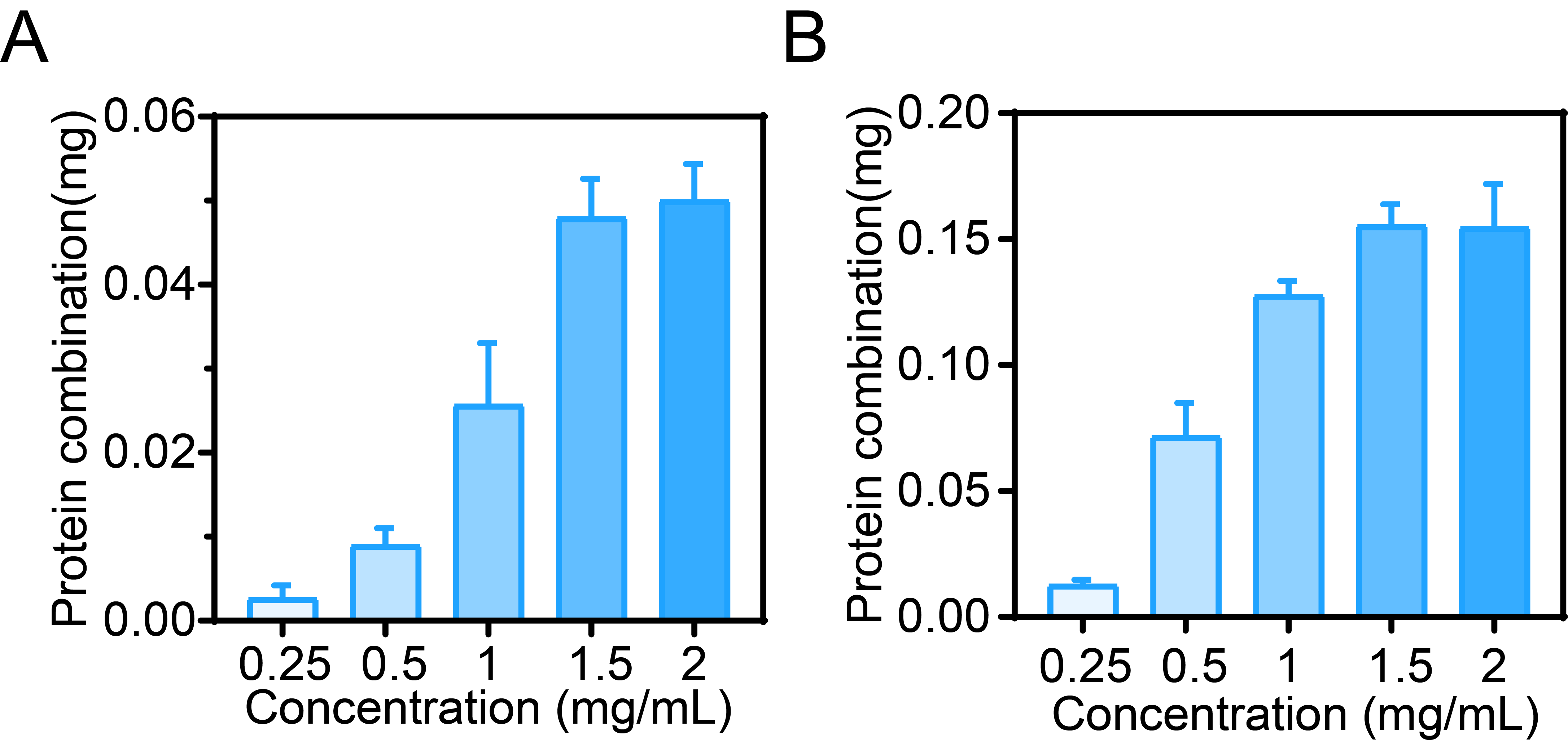


Figure S1. Encapsulation of Cas9/sgRNA by A) CSN-COOH, B) CSN-PEI.


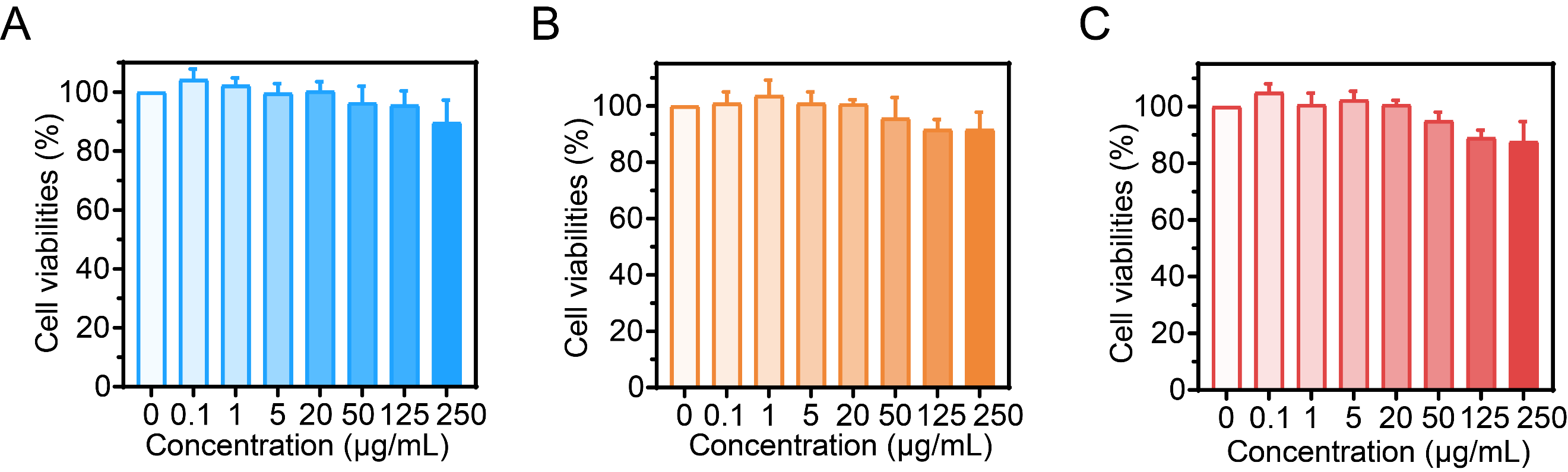


Figure S2. Cell viability of HPDE6C7 48h after CSN, CSN-COOH or CSN-PEI treatment.


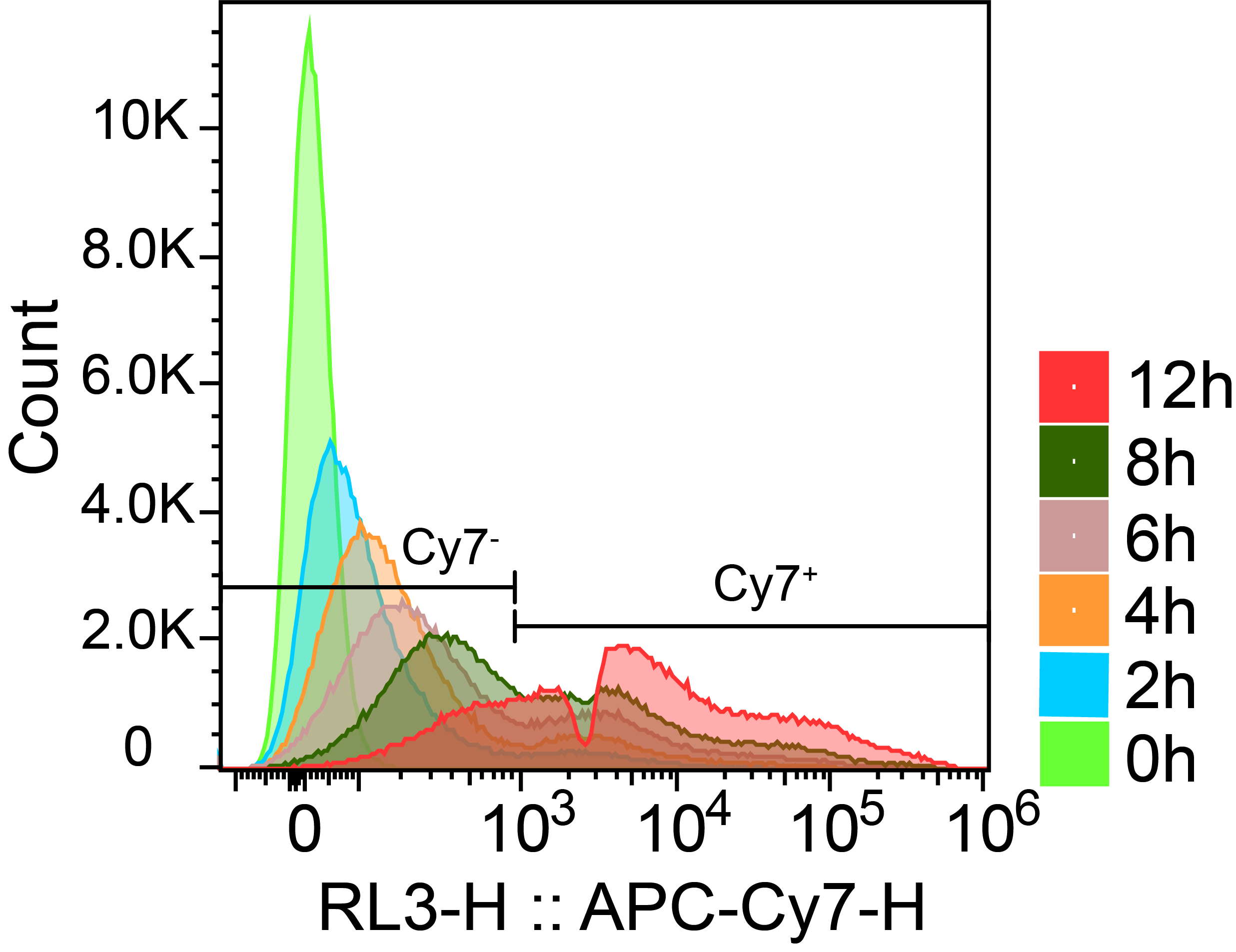


Figure S3. Internalization kinetics of TriCON in cells analyzed by flow cytometry. The histograms show the distribution of fluorescence intensity in cells after co-incubation with Cy7-fluorescently labeled TriCON for different times (0h, 2h, 4h, 6h, 8h, 12h).


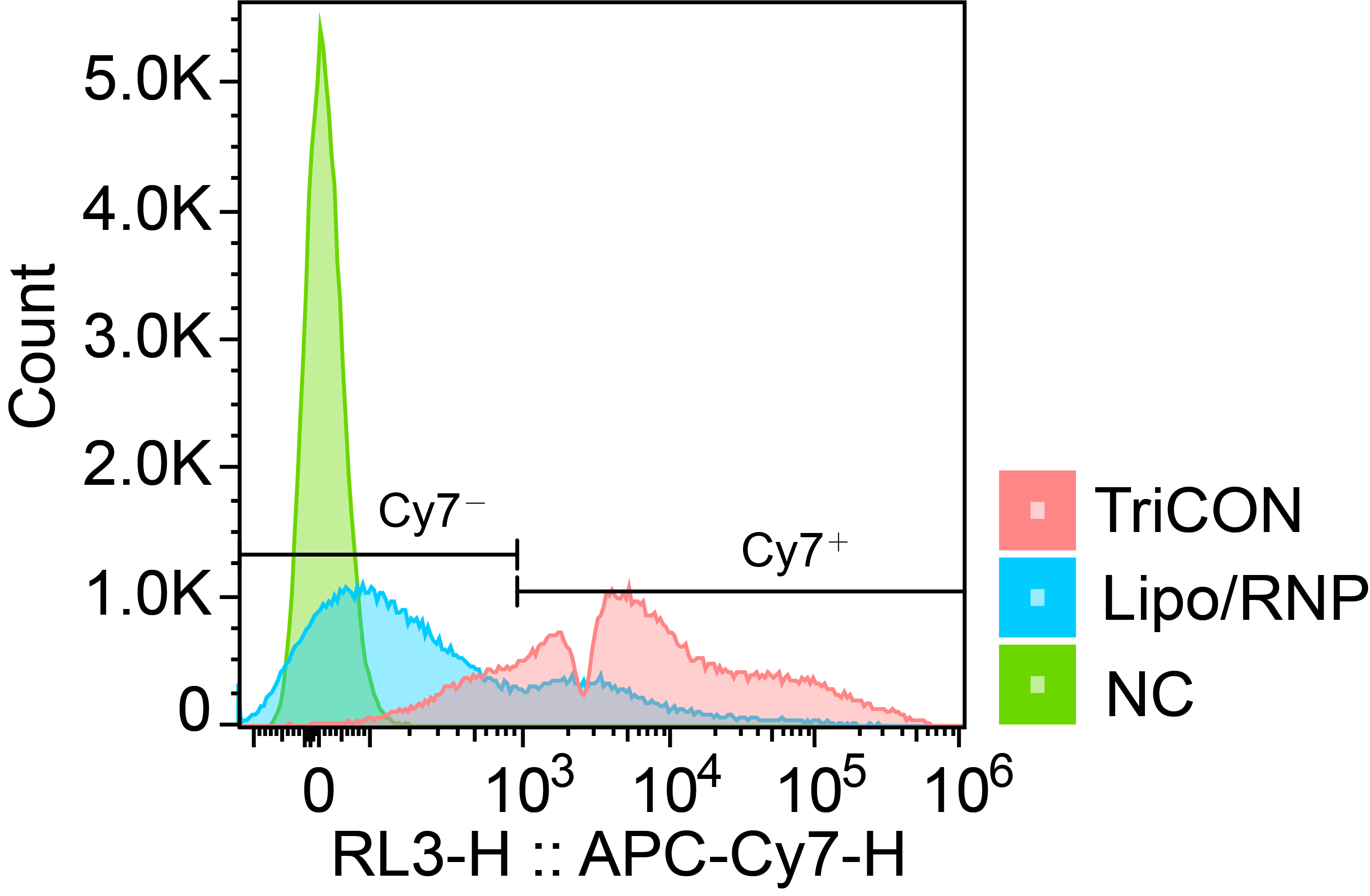


Figure S4. Internalization kinetics of TriCON and Lipo/RNP in cells analyzed by flow cytometry. The histograms show the fluorescence intensity of cells after co-incubation with Cy7-labeled TriCON and liposome-encapsulated RNP for 12 hours, respectively.


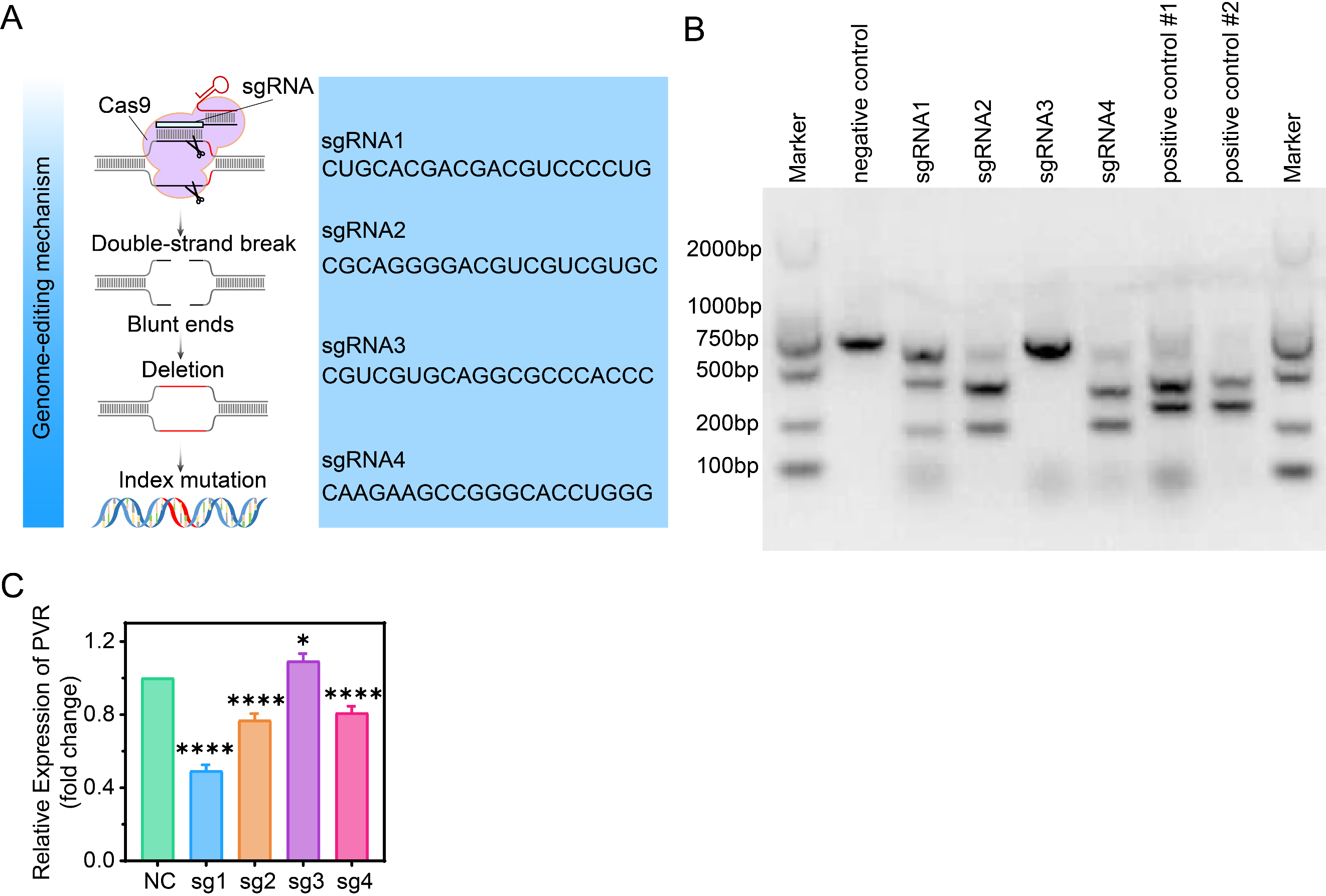


Figure S5. Knockdown of PVR gene. A) Schematic diagram of the working principle of CRISPR/Cas9. B) T7E1 detection after knockout of PVR by different sgRNAs. C) Real-time fluorescence quantitative PCR detection of PVR expression in pancreatic cancer cells treated with different sgRNAs.


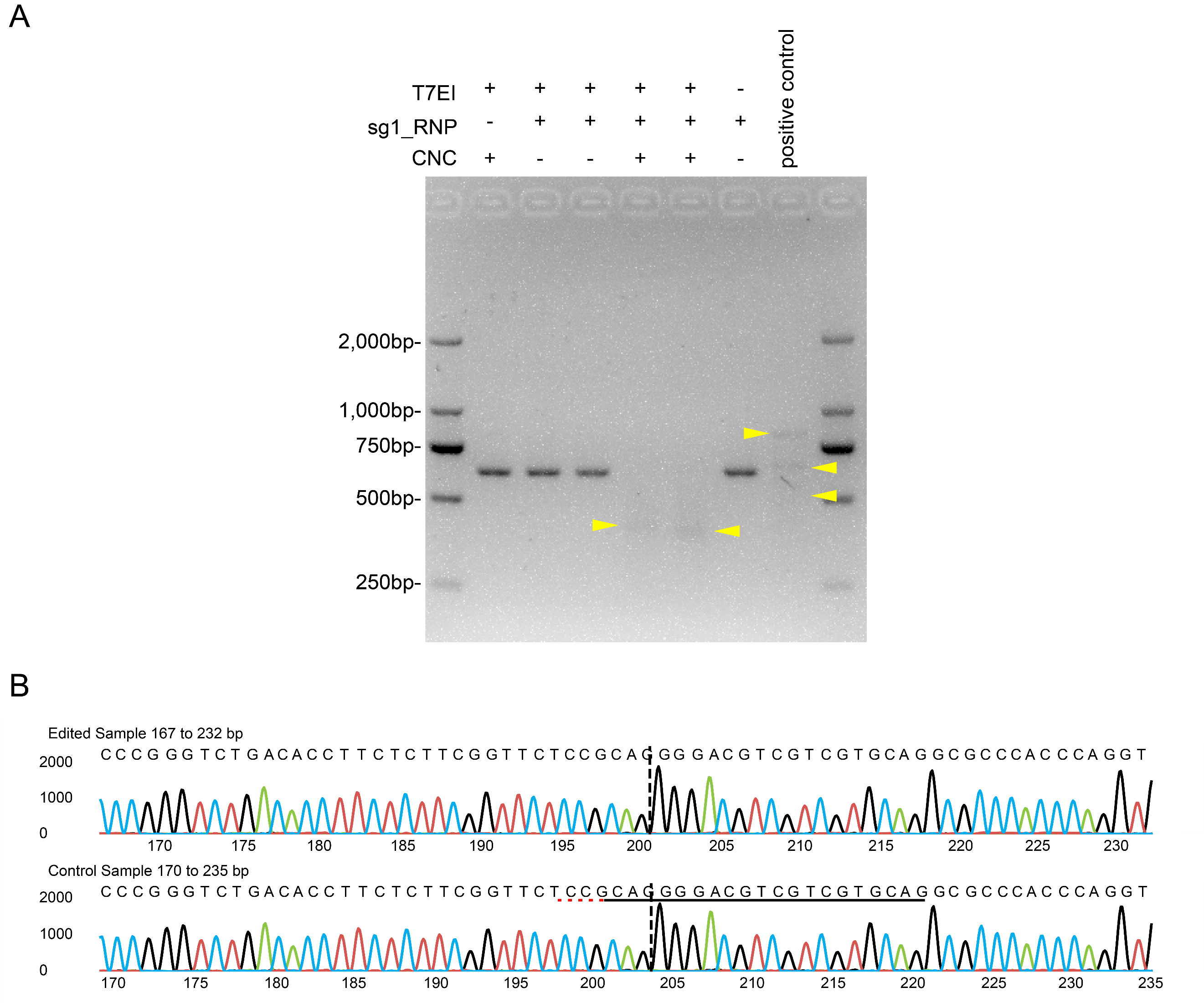


Figure S6. The knockout efficiency of the CRISPR system in cells. A) T7E1 detection after knockout of PVR by TriCON. B) Sanger sequencing after negative control treatment.


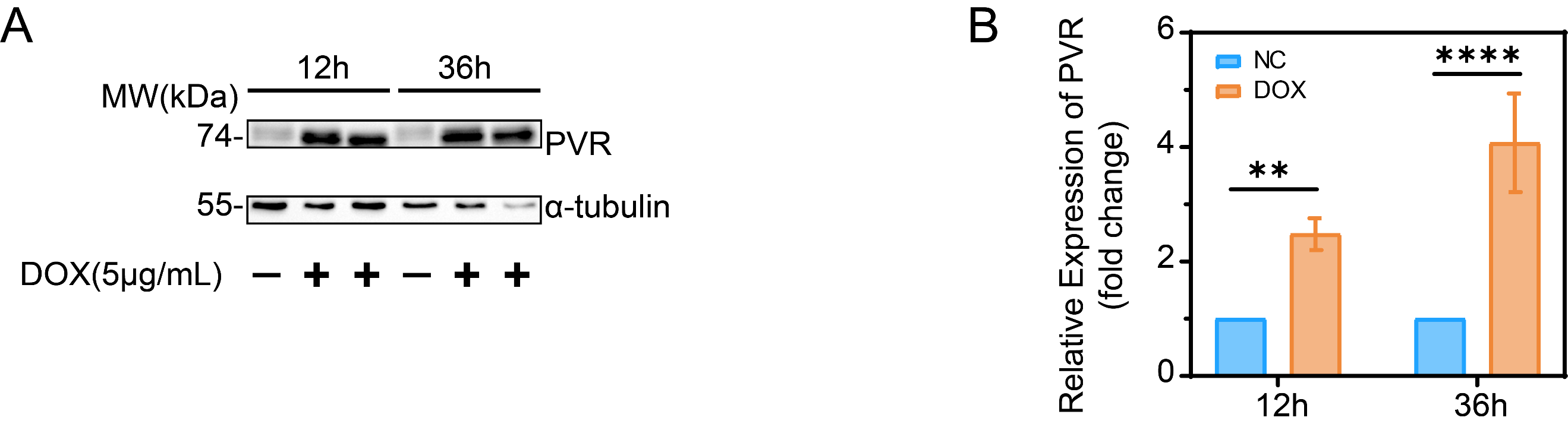


Figure S7. Expression(A) and quantification(B) of PVR in DOX-treated PDAC cells. The data in (B) are presented as mean ± SD and from n = 3 biologically independent samples. Statistical significance was assessed using two‐tailed Student's t‐tests. (**P < 0.01, ****P < 0.0001).


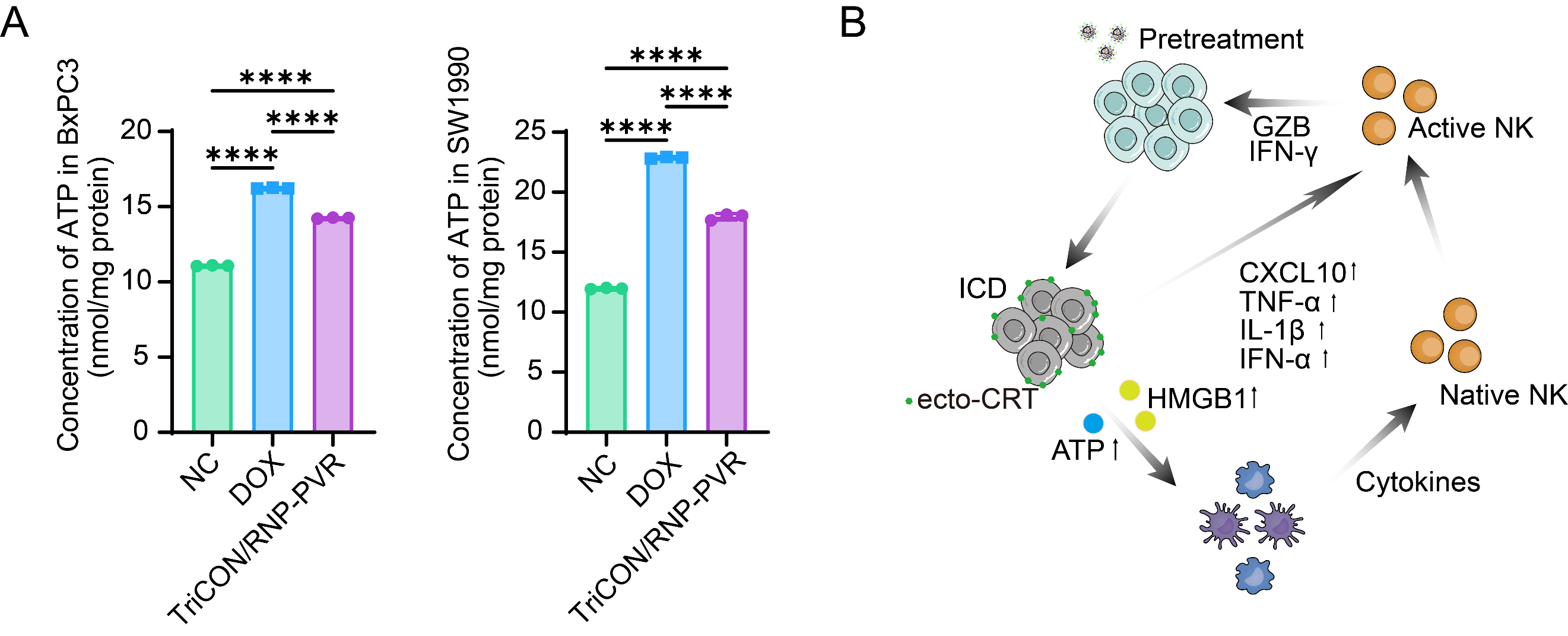


Figure S8. A) Quantification of intracellular ATP in BxPC3 and SW1990 after DOX and TriCON/RNP-PVR treatment. B) Schematic diagram illustrating tumor destruction by enhanced NK cells through immunogenic cell death. Data are presented as mean ± SD and from n = 3 biologically independent samples. Statistical significance was assessed using one-way analysis of variance (ANOVA). (****P < 0.0001).


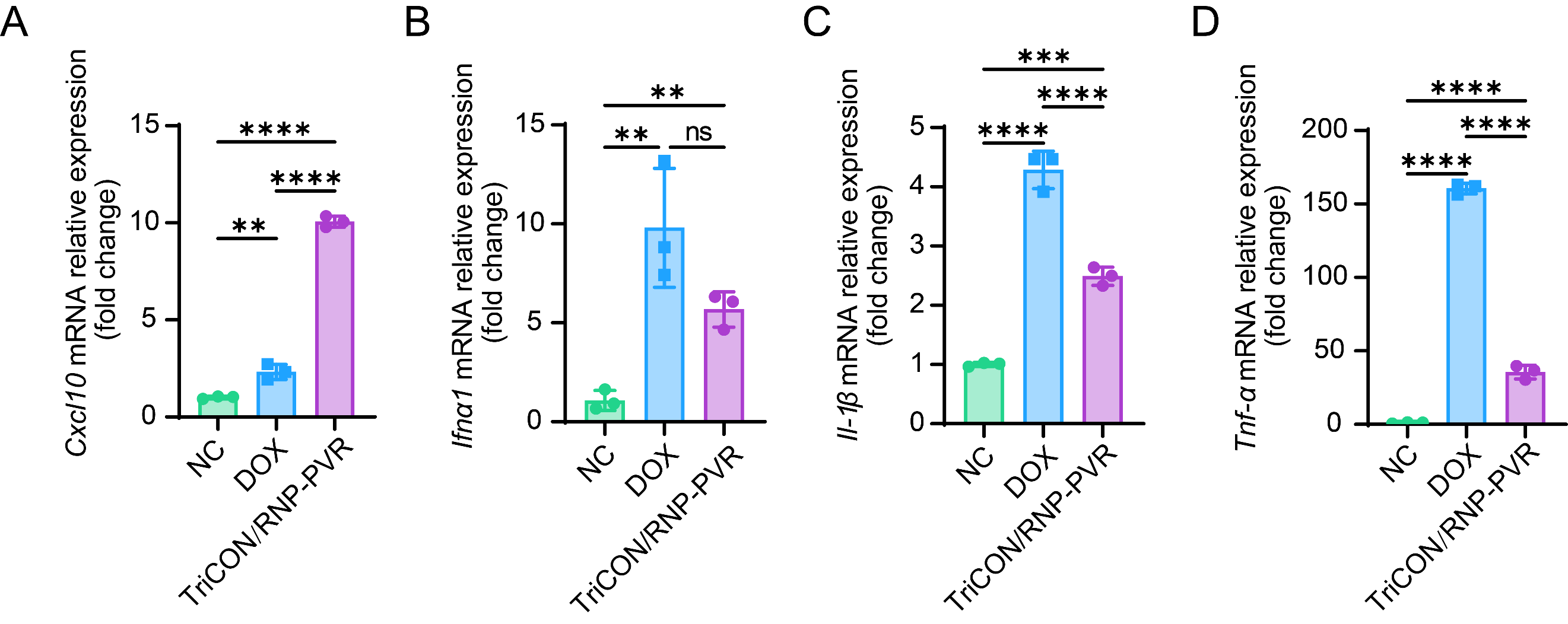


Figure S9. mRNA levels of CXCL10, IFNα1, IL-1β, TNF-α in tumor cells after DOX and TriCON/RNP-PVR treatment. All data are presented as mean ± SD and from n = 3 biologically independent samples. Statistical significance was assessed using one-way analysis of variance (ANOVA). (ns for P > 0.05, **P < 0.01, ***P < 0.001, ****P < 0.0001).


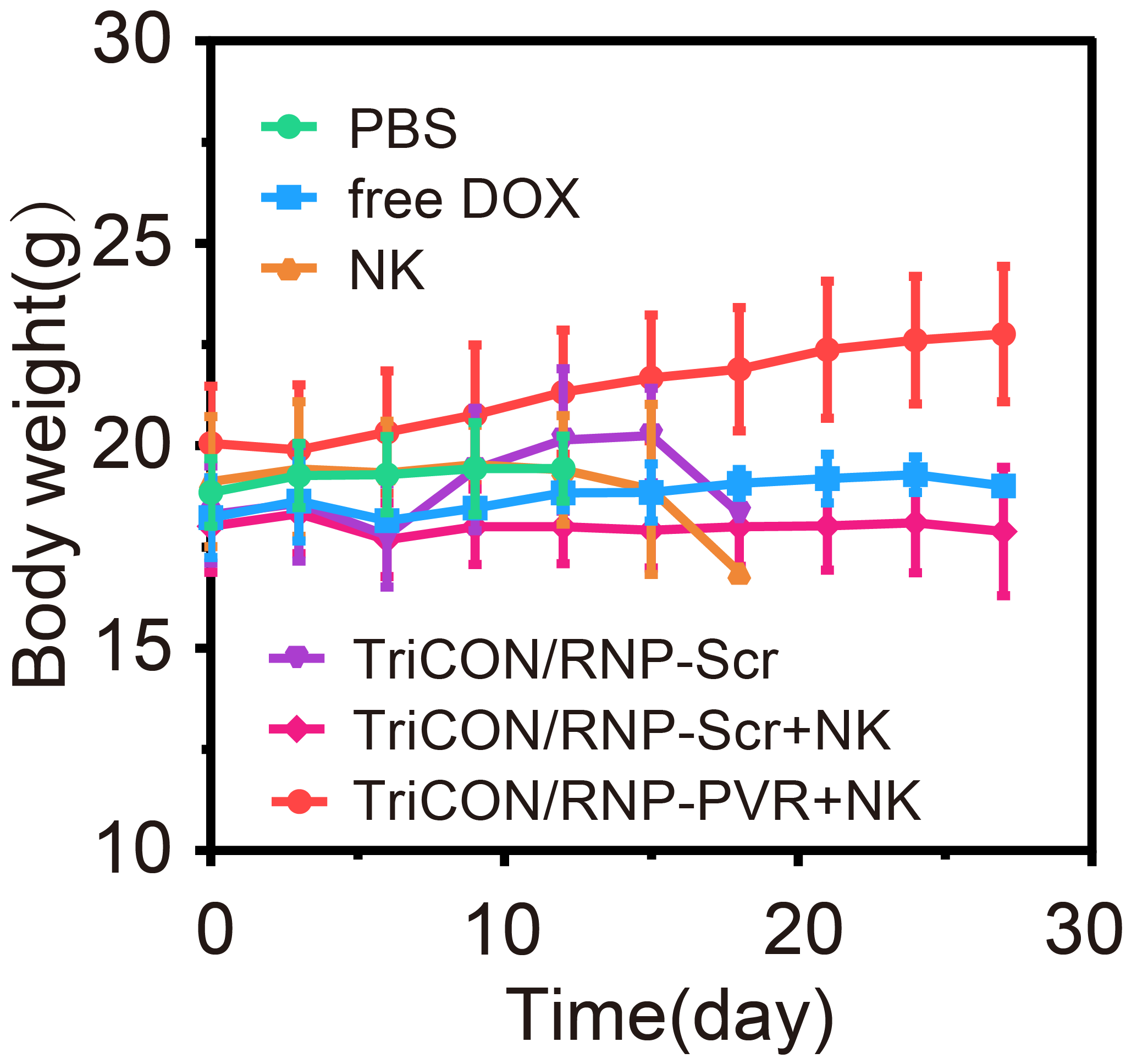


Figure S10. Changes in body weight after treating the mice with different formulations.


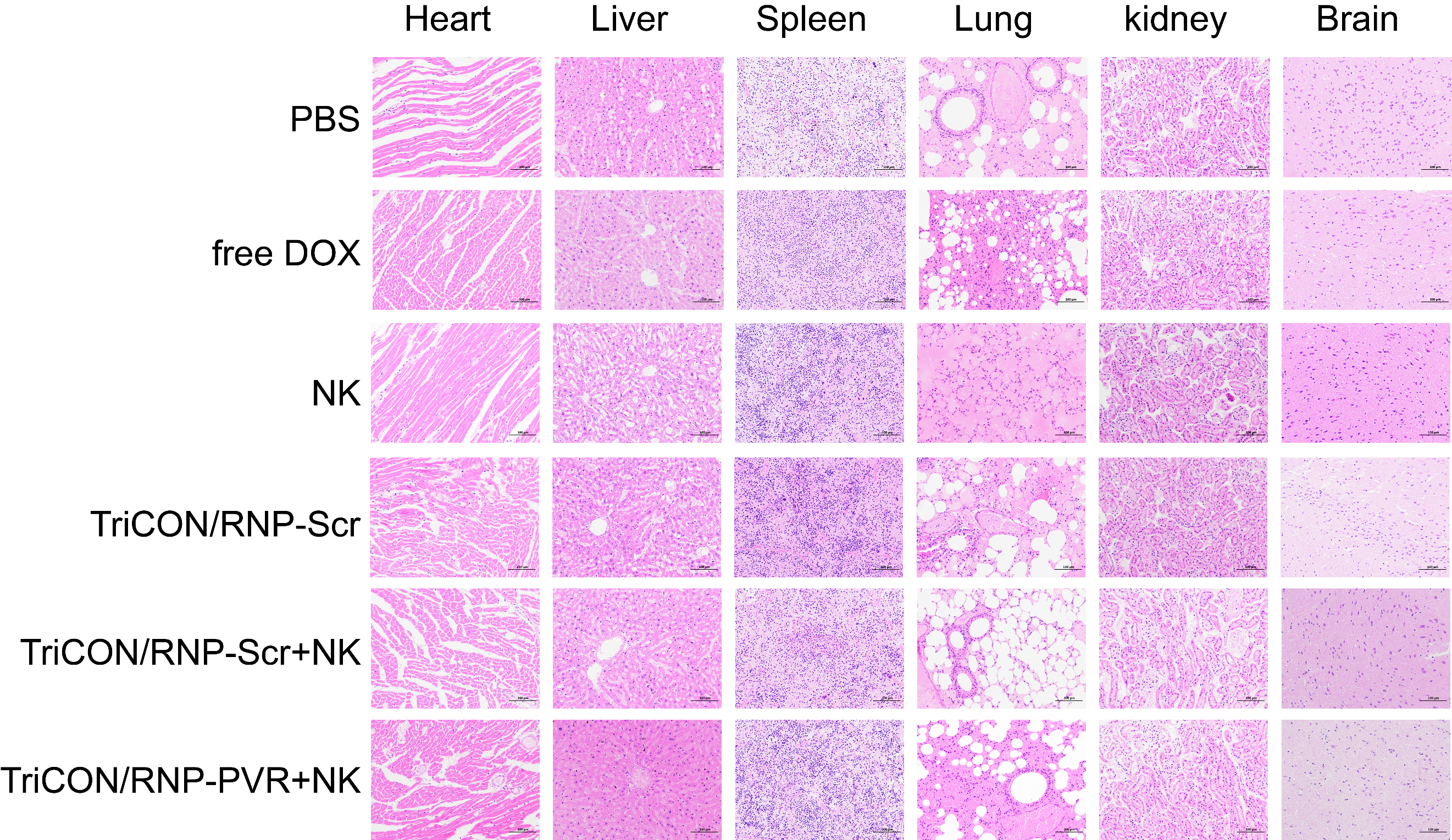


Figure S11. Corresponding H&E stained pathological sections of each organ. Scale bar: 100 μm.


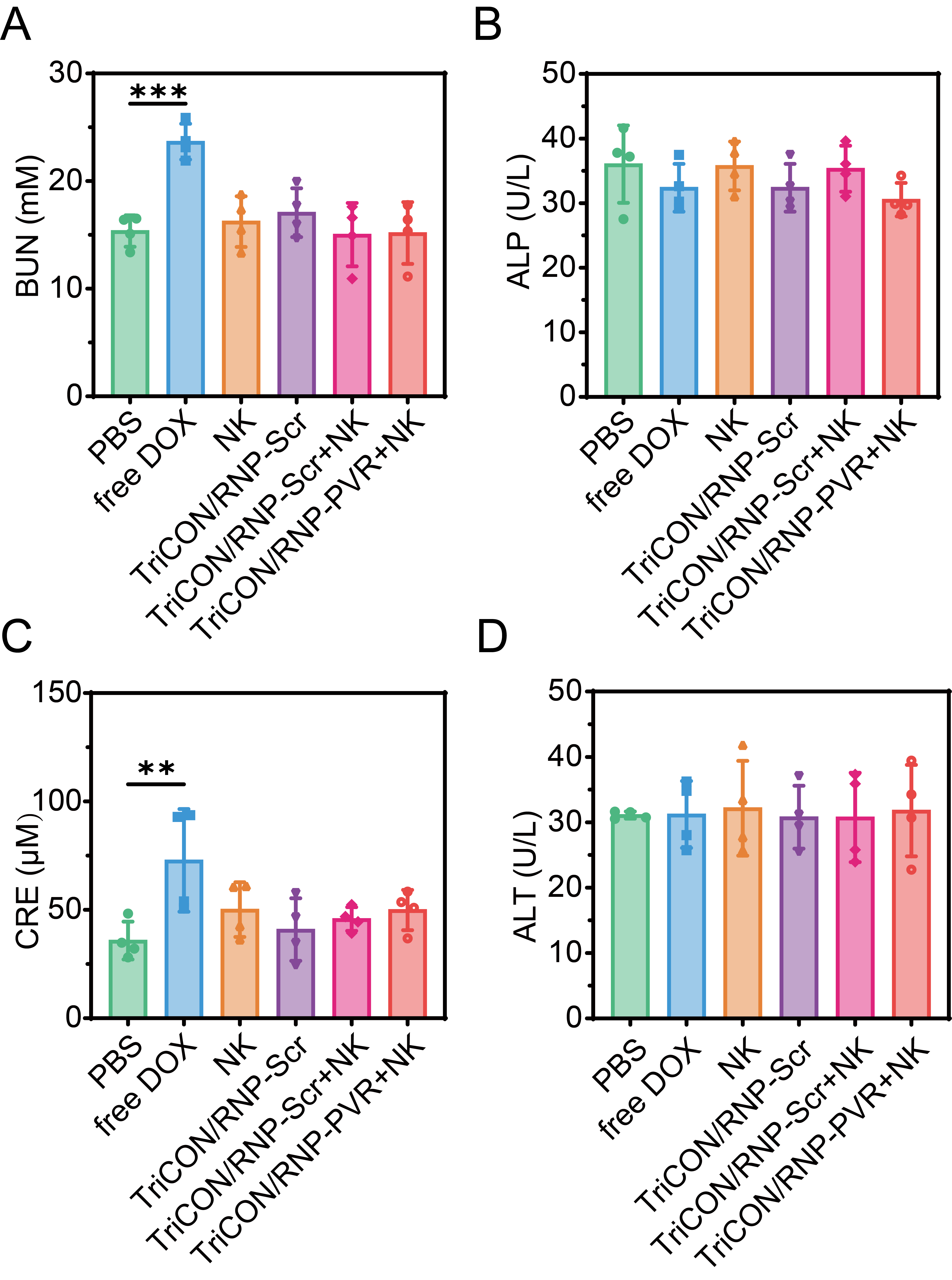


Figure S12. Blood biochemical analysis of mice after intravenous injection of each drug (n = 4). Statistical significance was assessed using one-way analysis of variance (ANOVA). (**P < 0.01, ***P < 0.001).


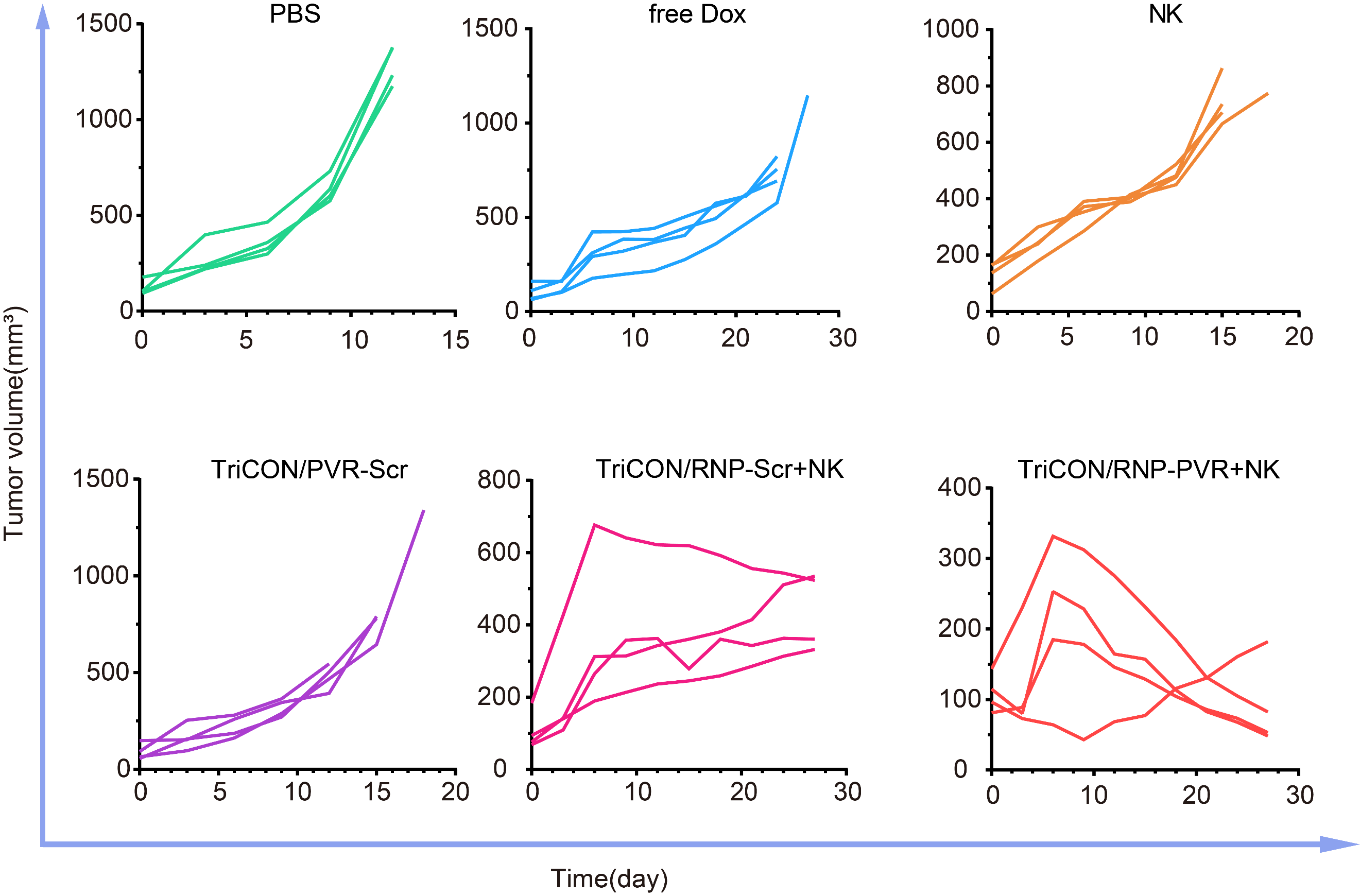


Figure S13. Individual growth curves of PDAC tumors in different treatment groups.


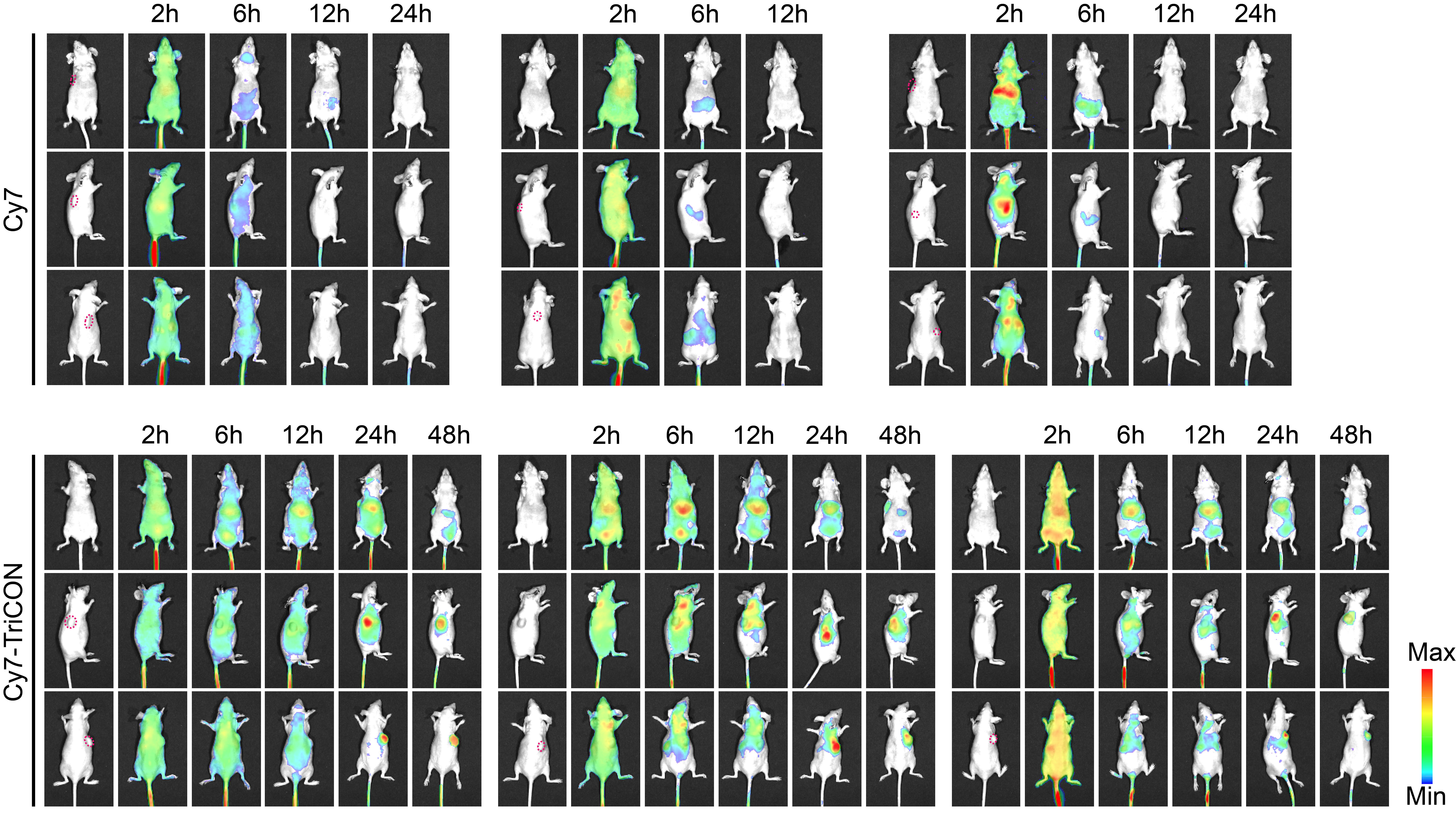


Figure S14. All in vivo fluorescence images used for quantitative analysis were captured in three positions per mouse: supine, prone, and lateral, with n=3.


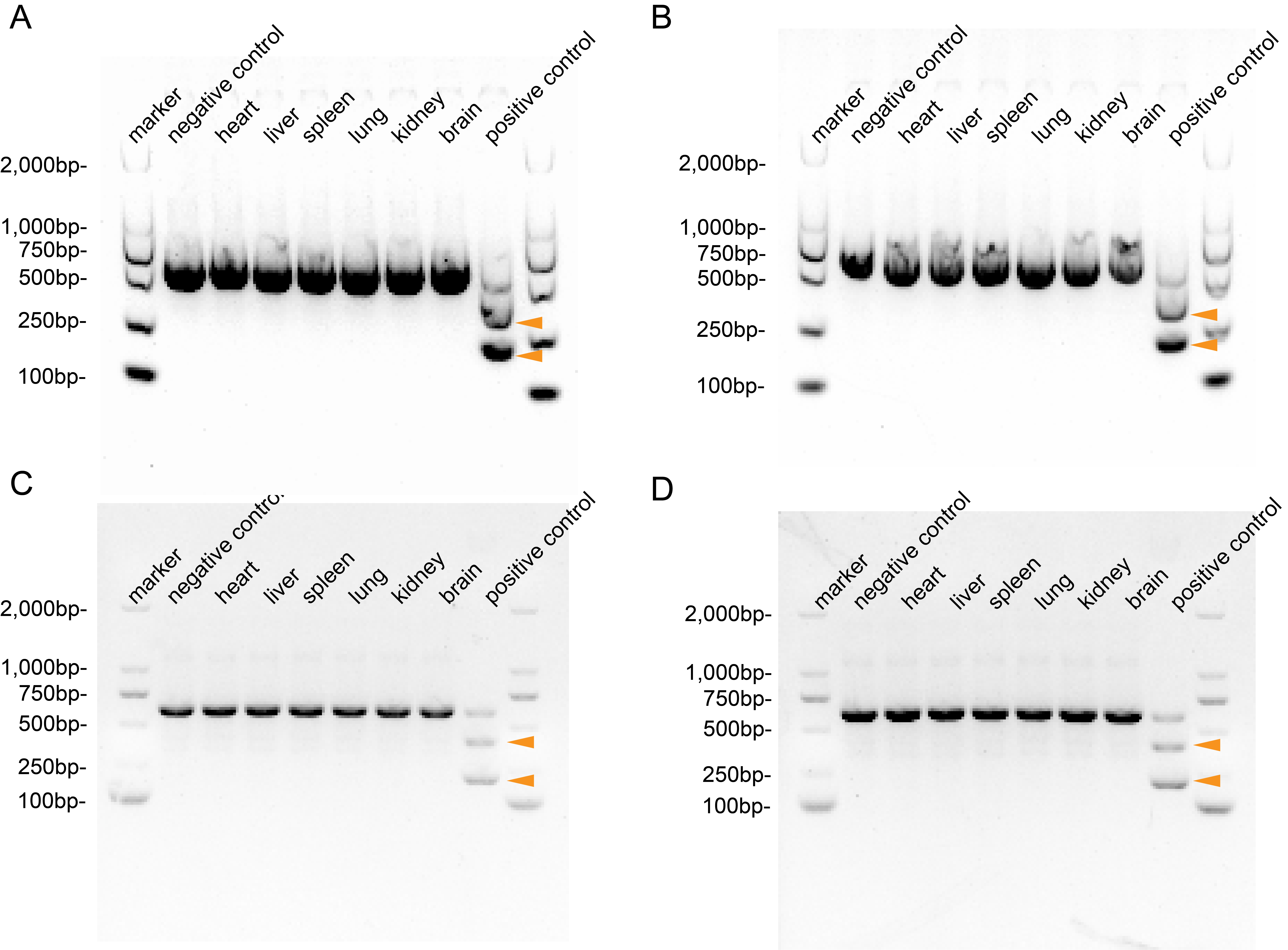


Figure S15. T7E1 assay of corresponding organ tissues in individual mice after TriCON/RNP-PVR treatment.


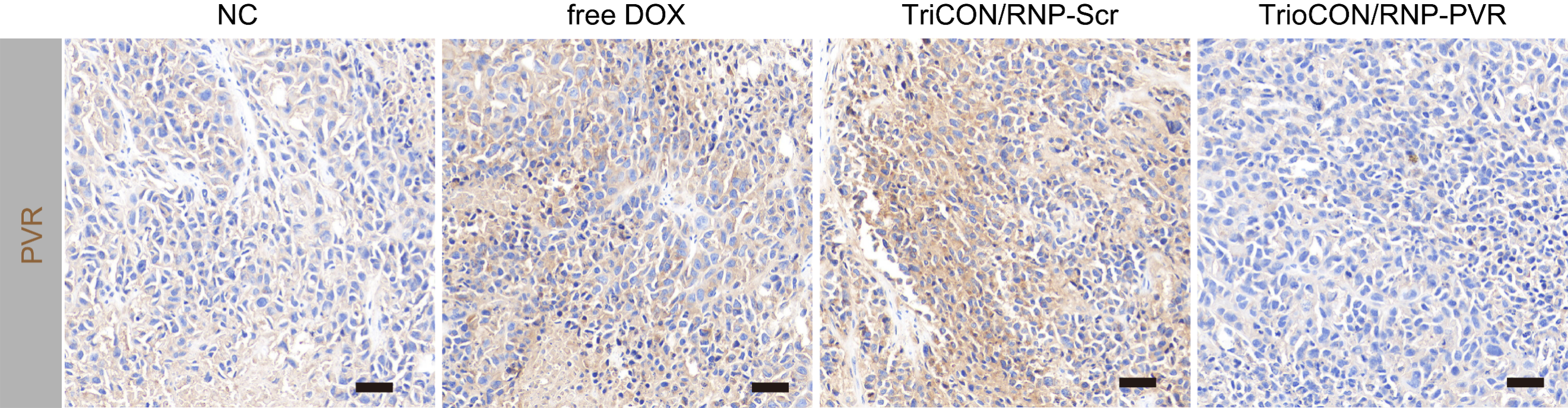


Figure S16. Representative PVR immunohistochemical staining images of tumor tissues from each treatment group (scale bar = 50μm)


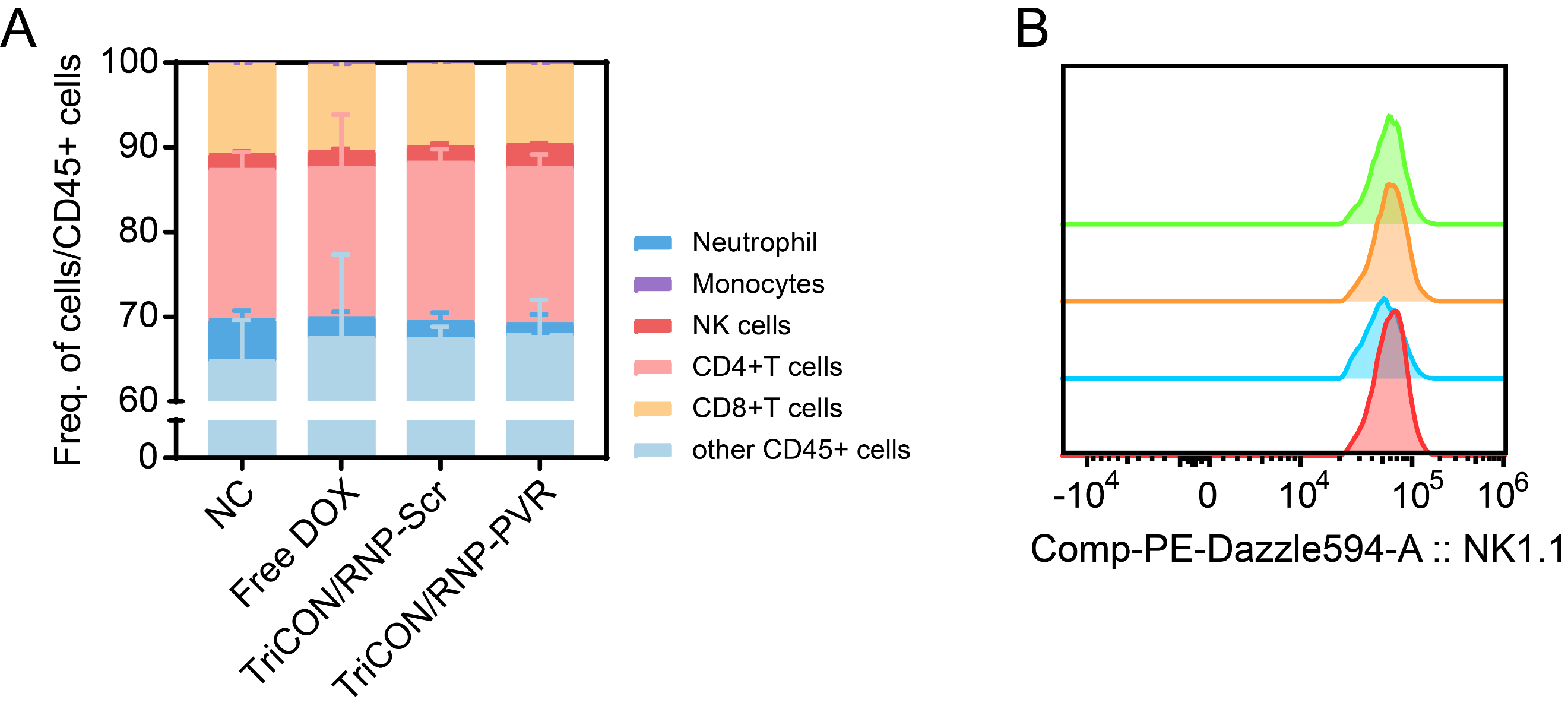


Figure S17. The effect of different treatments on NK cell infiltration in the tumor immune microenvironment. A) Flow cytometry analysis revealed the percentage distribution of each immune cell subset among CD45-positive leukocytes in tumor tissues. B) Representative histogram of NK cell marker NK1.1 detected by flow cytometry.


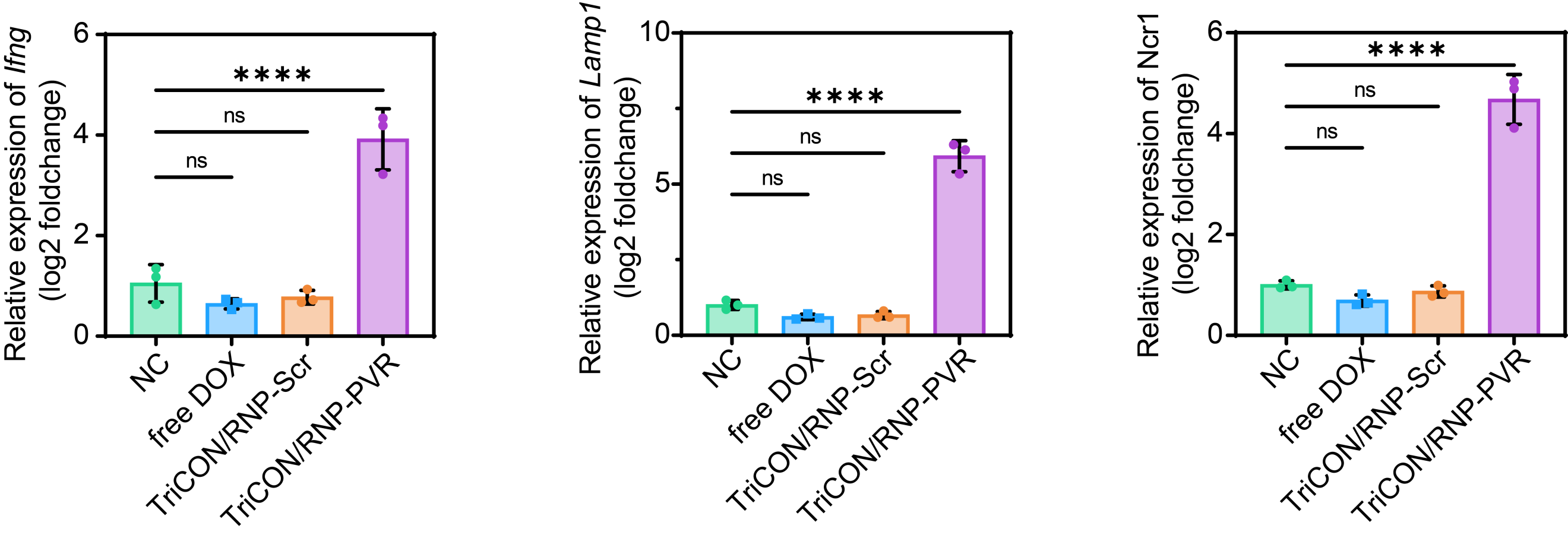


Figure S18. The relative expression levels of IFN-γ, LAMP1, and NCR1 mRNA in tumor tissues from different treatment groups. Data are presented as mean ± SD. Statistical significance was assessed using one-way analysis of variance (ANOVA). (ns for P > 0.05, ****P < 0.0001).


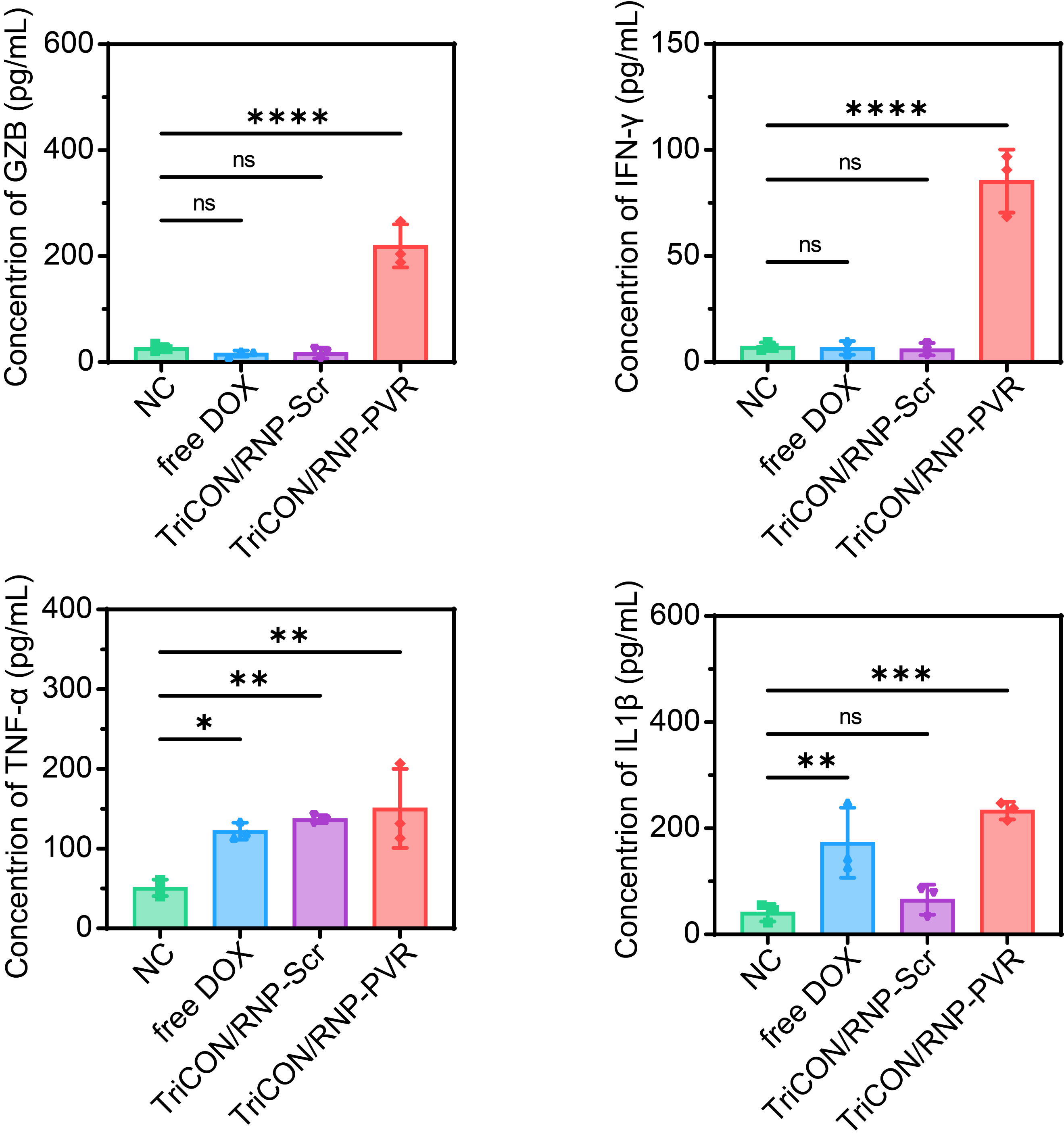


Figure S19. GZB, IFN-γ, TNF-α and IL-1β levels in tumors from mice treated with different formulations. All data are presented as mean ± SD. Statistical significance was assessed using one-way analysis of variance (ANOVA). (*P < 0.05, **P < 0.01, ***P < 0.001, ****P < 0.0001).


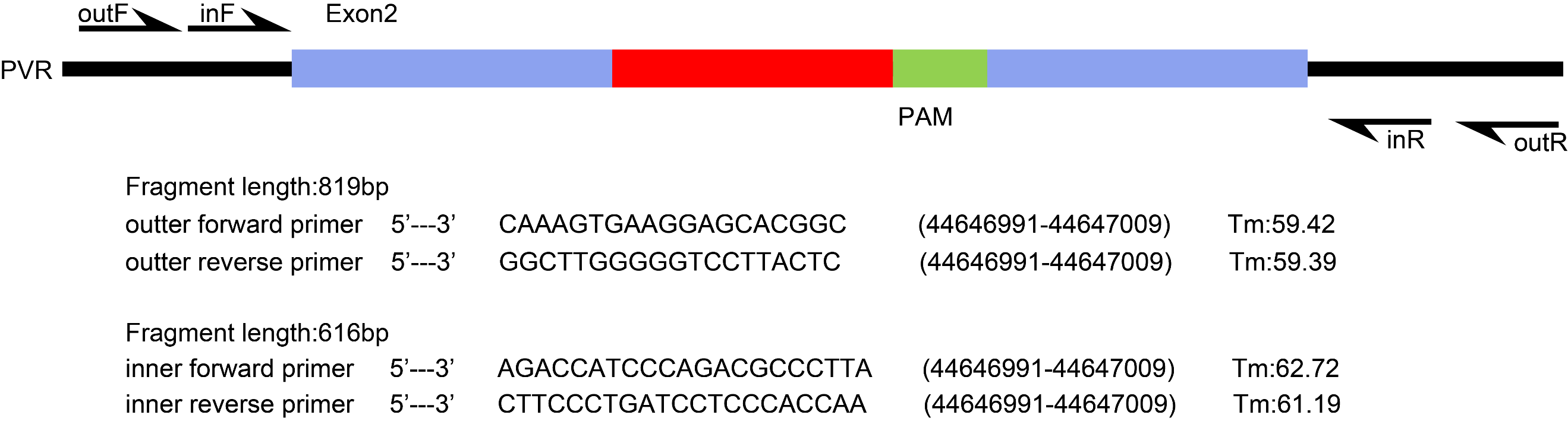


Figure S20. Schematic of genomic DNA amplification and primer sequences for PVR.


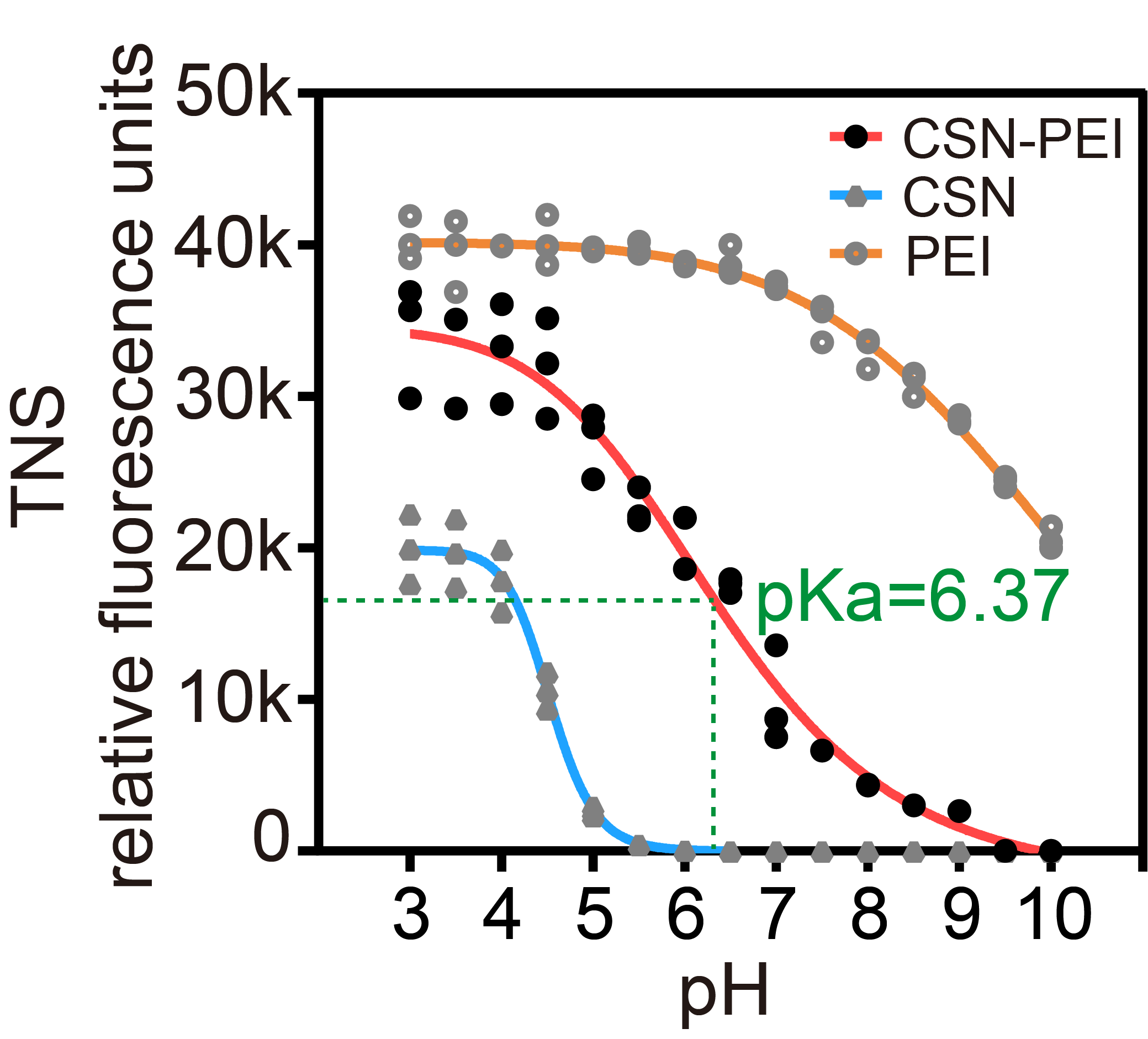


Figure S21. The apparent pKa values of CSN, PEI, and the CSN-PEI complex were determined using the TNS relative fluorescence unit method.


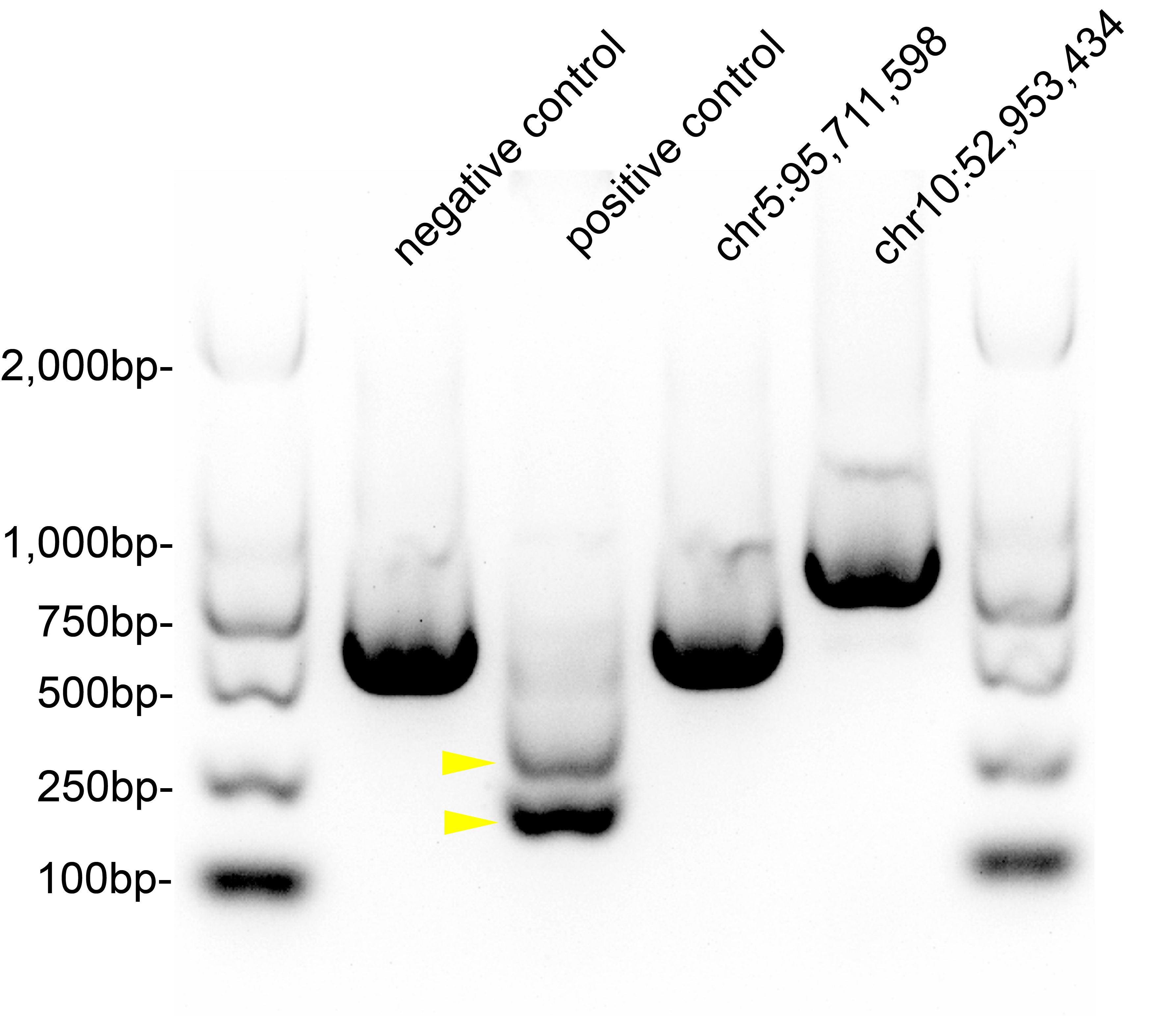


Figure S22. Assessment of off-target effects after TriCON/RNP-PVR treatment.


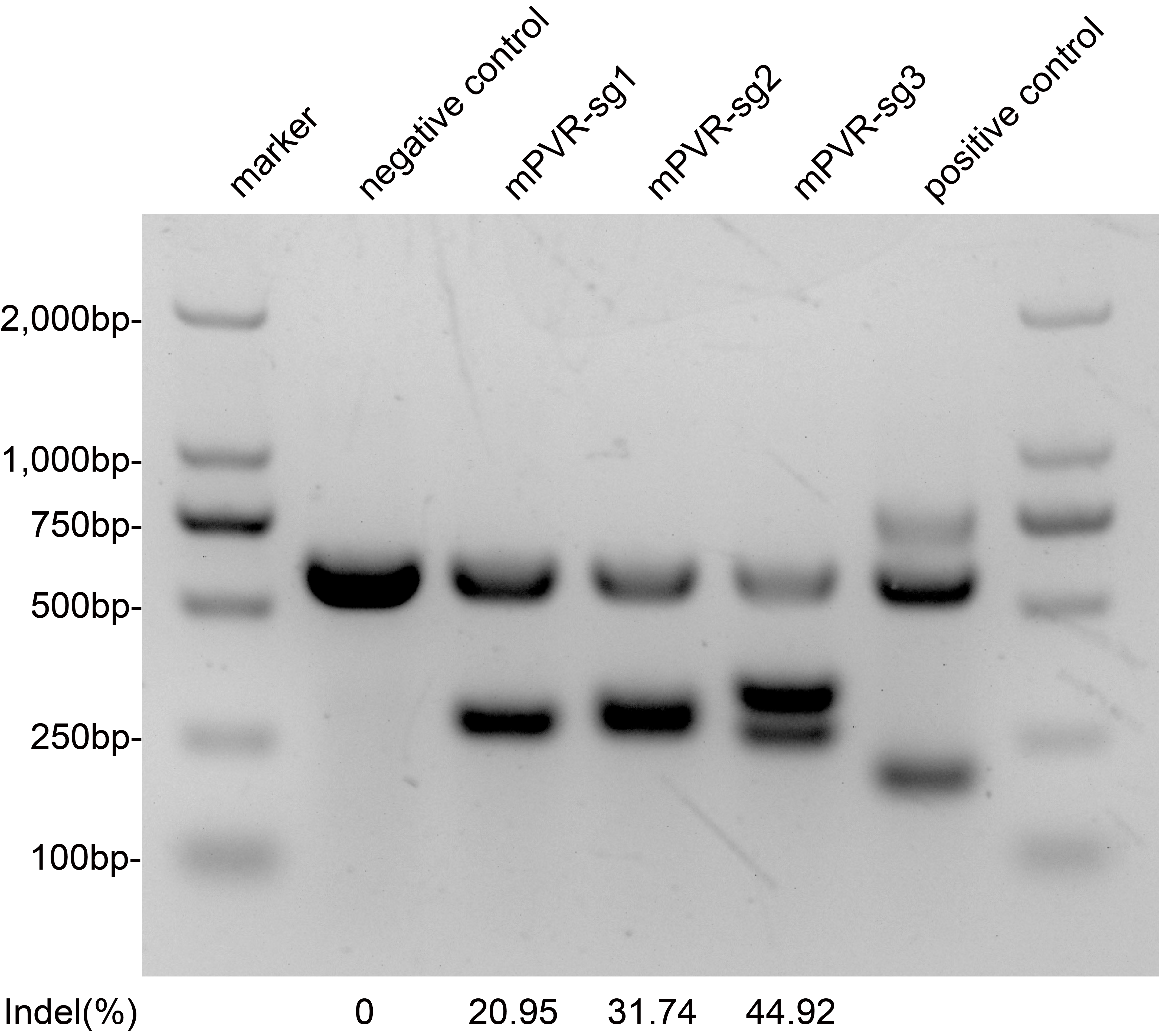


Figure S23. T7E1 results following knockout of mouse PVR by different sgRNAs.


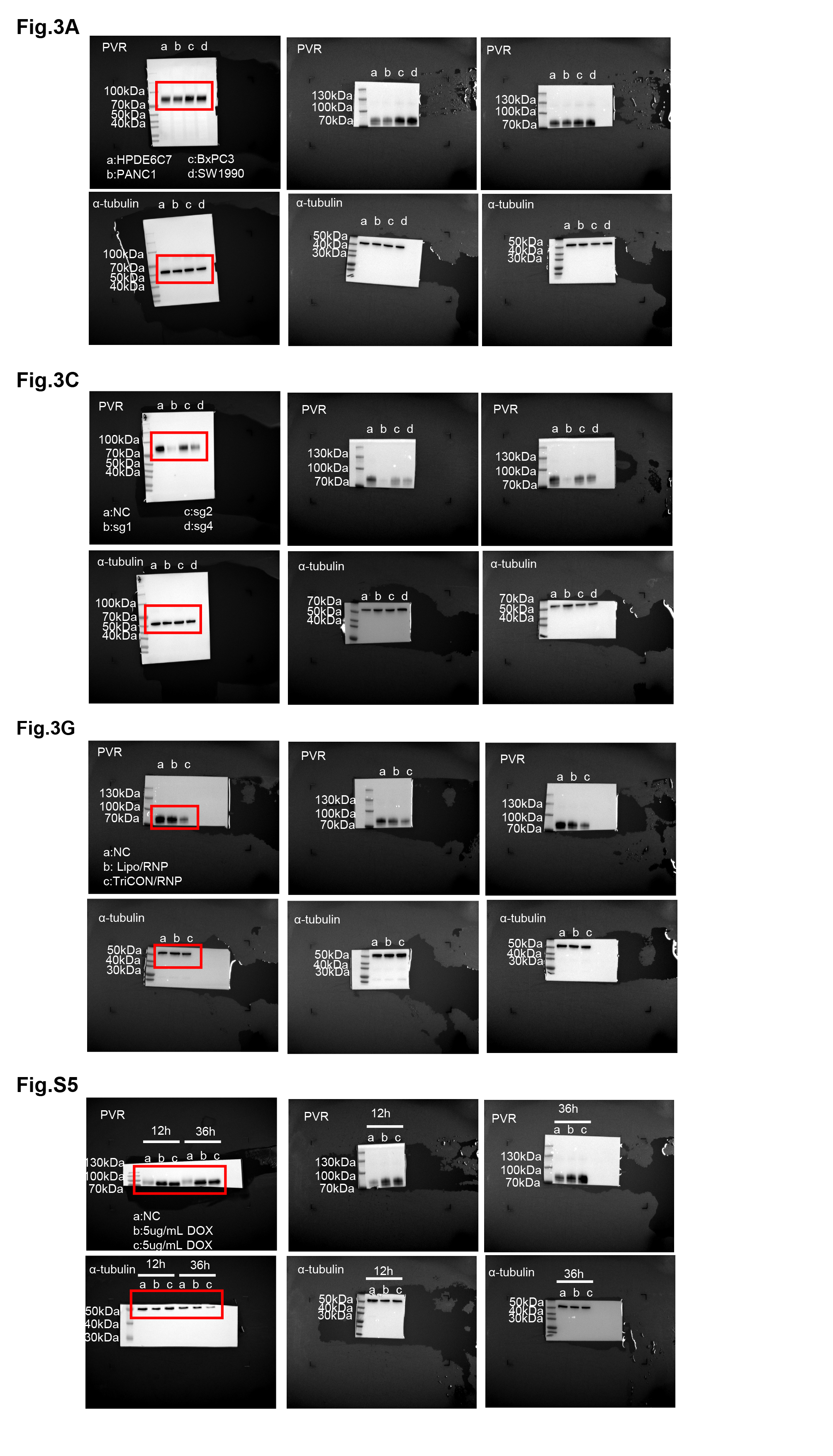


Figure S24. The supplementary Western blot images utilized in the quantitative analysis.

Table S1. All the antibodies used in the experiment.

| **Antibody Name** | **Manufacture (catalogue number)** | **Origin** | **Applications (working dilution)** |
| --- | --- | --- | --- |
| anti-α-tubulin | HuaBio (HA721913) | Rabbit | WB (1:2000) |
| anti-PVR | Cell Signaling Technology (#81254) | Rabbit | WB (1:1000);  IHC (1:200) |
| anti-EEA1 | Cell Signaling Technology (#2411) | Rabbit | IF (1:100) |
| anti-HMGB1 | Proteintech (10829-1-AP) | Rabbit | WB (1:5000) |
| anti-Histone H3 | Beyotime (AF0009) | Mouse | WB (1:1000) |
| anti-rabbit IgG-HRP | Beyotime (A0208) | Goat | WB (1:3000) |
| anti-mouse IgG-HRP | Beyotime (A0216) | Goat | WB (1:3000) |
| anti-Rabbit IgG-488 | Invitrogen (A-11008) | Goat | IF (1:5000) |
| anti-Ki67 | Servicebio (GB151141) | Mouse | IHC (1:150) |
| anti-Mouse IgG-488 | Proteintech (SA00013-1) | Goat | IF (1:1500) |
| anti-RAB7A | Proteintech (84741-1-RR) | Rabbit | IF (1:400) |
| anti-LAMP1 | Cell Signaling Technology (# 15665) | Mouse | IF (1:50) |
| anti-CD11b | Biolegend (#101242) | Rat | FCM (1:100) |
| anti-CD45 | Biolegend (103127) | Rat | FCM (1:100) |
| anti-CD3 | Biolegend (100271) | Rat | FCM (1:100) |
| anti-LY6G | Biolegend (127613) | Rat | FCM (1:100) |
| anti-CD4 | Biolegend (100542) | Rat | FCM (1:100) |
| anti-CD8a | Biolegend (100750) | Rat | FCM (1:100) |
| anti-NK1.1 | Biolegend (108747) | Mouse | FCM (1:100) |
| anti-CRT | Proteintech (10292-1-AP) | Rabbit | IF (1:250)  FCM (1:100) |

Table S2. The PDI index of TriCON particle size measured by dynamic light scattering under varying conditions and time intervals.

| Time(d) | PDI index | | |
| --- | --- | --- | --- |
|  | PBS | 1640 | 1640 + 10% FBS |
| 0 | 0.280 ± 0.001 | 0.283 ± 0.003 | 0.273 ± 0.004 |
| 3 | 0.283 ± 0.002 | 0.276 ± 0.001 | 0.291 ± 0.005 |
| 7 | 0.284 ± 0.005 | 0.281 ± 0.002 | 0.206 ± 0.002 |
| 14 | 0.288 ± 0.003 | 0.290 ± 0.002 | 0.277 ± 0.003 |
| 21 | 0.288 ± 0.003 | 0.275 ± 0.001 | 0.297 ± 0.005 |
| 28 | 0.290 ± 0.002 | 0.279 ± 0.002 | 0.283 ± 0.005 |

Table S3. Primer sequences for PCR in this study.

| Primer names | Sequences (5'-3') | Application |
| --- | --- | --- |
| hGAPDH-F | GACAGTCAGCCGCATCTTCT | qPCR |
| hGAPDH-R | GCGCCCAATACGACCAAATC | qPCR |
| mGAPDH-F | CATCACTGCCACCCAGAAGACTG | qPCR |
| mGAPDH-R | ATGCCAGTGAGCTTCCCGTTCAG | qPCR |
| hPVR-F | CCGTCCAGGTCAAAGGTACAG | qPCR |
| hPVR-R | TCACCTTGTGCCCTCTGTCT | qPCR |
| mPVR-JD-F | TTACGAGCTACTCAGGGAATG | PCR |
| mPVR-JD-R | ACACTTACCTTGCACCTTCA | PCR |
| hCXCL10-F | GTGGCATTCAAGGAGTACCTC | qPCR |
| hCXCL10-R | TGATGGCCTTCGATTCTGGATT | qPCR |
| hIL17-F | CGGACTGTGATGGTCAACCTGA | qPCR |
| hIL17-R | GCACTTTGCCTCCCAGATCACA | qPCR |
| hIL1β-F | CCACAGACCTTCCAGGAGAATG | qPCR |
| hIL1β-R | GTGCAGTTCAGTGATCGTACAGG | qPCR |
| hIL6-F | AGACAGCCACTCACCTCTTCAG | qPCR |
| hIL6-R | TTCTGCCAGTGCCTCTTTGCTG | qPCR |
| hTNFα-F | CTCTTCTGCCTGCTGCACTTTG | qPCR |
| hTNFα-R | ATGGGCTACAGGCTTGTCACTC | qPCR |
| PVR-sg1F | TTAATACGACTCACTATAGGGCTGCACGACGACGTCCCCTGGTTTTAGAGCTAGAAATA | in vitro transcription |
| mIFN-γ-F | CAGCAACAGCAAGGCGAAAAAGG | qPCR |
| mIFN-γ-R | TTTCCGCTTCCTGAGGCTGGAT | qPCR |
| mLAMP1-F | CCAGGCTTTCAAGGTGGACAGT | qPCR |
| mLAMP1-R | GGTAGGCAATGAGGACGATGAG | qPCR |
| mNCR1-F | TAGGGCTCACAGAGGGACATAC | qPCR |
| mNCR1-R | GTAGGTGCAAGGCTGCTGTTCT | qPCR |
| hPVR-sg2F | TTAATACGACTCACTATAGGGCGCAGGGGACGTCGTCGTGCGTTTTAGAGCTAGAAATA | in vitro transcription |
| hPVR-sg3F | TTAATACGACTCACTATAGGGCGTCGTGCAGGCGCCCACCCGTTTTAGAGCTAGAAATA | in vitro transcription |
| hPVR-sg4F | TTAATACGACTCACTATAGGGCAAGAAGCCGGGCACCTGGGGTTTTAGAGCTAGAAATA | in vitro transcription |
| mPVR-sg1F | TTAATACGACTCACTATAGGGATTCGACAGGCGTCTTGGGAGTTTTAGAGCTAGAAATA | in vitro transcription |
| mPVR-sg2F | TTAATACGACTCACTATAGGGAATTCGACAGGCGTCTTGGGGTTTTAGAGCTAGAAATA | in vitro transcription |
| mPVR-sg3F | TTAATACGACTCACTATAGGGAAGACGCCTGTCGAATTGTAGTTTTAGAGCTAGAAATA | in vitro transcription |
| Chr5_outerF | ATTCCACCTCCAAAGTCCTC | PCR |
| Chr5_outerR | CACTTGAGGAATTTGGGTCC | PCR |
| Chr5_innerF | CACTTGAGGAATTTGGGTCC | PCR |
| Chr5_innerR | CACTTGAGGAATTTGGGTCC | PCR |
| Chr10_outerF | TTGATACCTGCAAGGAGATACC | PCR |
| Chr10_outerR | AGAAGGGTCACTGAATCTCAC | PCR |
| Chr10_innerF | CTCTTTGGATACGGTCCC | PCR |
| Chr10_innerR | CTTAAAAAGGGCAGACTCTGG | PCR |

**References:**

[1] H. Zhang, O. Noonan, X. Huang, Y. Yang, C. Xu, L. Zhou, C. Yu, *ACS Nano* **2016**, *10* (4), 4579, <https://doi.org/10.1021/acsnano.6b00723>.

[2] X. Li, Y. Yan, Y. Lin, J. Jiao, D. Wang, D. Di, Y. Zhang, T. Jiang, Q. Zhao, S. Wang, *Journal of Colloid and Interface Science* **2017**, *494*, 159, <https://doi.org/10.1016/j.jcis.2017.01.090>.

[3] D. Y. Guschin, A. J. Waite, G. E. Katibah, J. C. Miller, M. C. Holmes, E. J. Rebar, *Methods Mol Biol* **2010**, *649*, 247, <https://doi.org/10.1007/978-1-60761-753-2_15>.

[4] K. Kyrylkova, S. Kyryachenko, M. Leid, C. Kioussi, *Methods Mol. Biol.* **2012**, *887*, 41, <https://doi.org/10.1007/978-1-61779-860-3_5>.
